# A Fine-Structure Map of Spontaneous Mitotic Crossovers in the Yeast *Saccharomyces cerevisiae*

**DOI:** 10.1371/journal.pgen.1000410

**Published:** 2009-03-13

**Authors:** Phoebe S. Lee, Patricia W. Greenwell, Margaret Dominska, Malgorzata Gawel, Monica Hamilton, Thomas D. Petes

**Affiliations:** Department of Molecular Genetics and Microbiology, Duke University Medical Center, Durham, North Carolina, United States of America; The University of North Carolina at Chapel Hill, United States of America

## Abstract

Homologous recombination is an important mechanism for the repair of DNA damage in mitotically dividing cells. Mitotic crossovers between homologues with heterozygous alleles can produce two homozygous daughter cells (loss of heterozygosity), whereas crossovers between repeated genes on non-homologous chromosomes can result in translocations. Using a genetic system that allows selection of daughter cells that contain the reciprocal products of mitotic crossing over, we mapped crossovers and gene conversion events at a resolution of about 4 kb in a 120-kb region of chromosome V of *Saccharomyces cerevisiae*. The gene conversion tracts associated with mitotic crossovers are much longer (averaging about 12 kb) than the conversion tracts associated with meiotic recombination and are non-randomly distributed along the chromosome. In addition, about 40% of the conversion events have patterns of marker segregation that are most simply explained as reflecting the repair of a chromosome that was broken in G1 of the cell cycle.

## Author Summary

Most higher organisms have two copies of several different types of chromosomes. For example, the human female has 23 pairs of chromosomes. Although the chromosome pairs have very similar sequences, they are not identical. Members of a chromosome pair can swap segments from one chromosome to the other; these exchanges are called “recombination.” Most previous studies of recombination have been done in cells undergoing meiosis, the process that leads to the formation of eggs and sperm (gametes). Recombination, however, can also occur in cells that are dividing mitotically. In our study, we examine the properties of mitotic recombination in yeast. We show that mitotic recombination differs from meiotic recombination in two important ways. First, the sizes of the chromosome segments that are non-reciprocally transferred during mitotic recombination are much larger than those transferred during meiotic exchange. Second, in meiosis, most recombination events involve the repair of a single chromosome break, whereas in mitosis, about half of the recombination events appear to involve the repair of two chromosome breaks.

## Introduction

**Figure 1:**
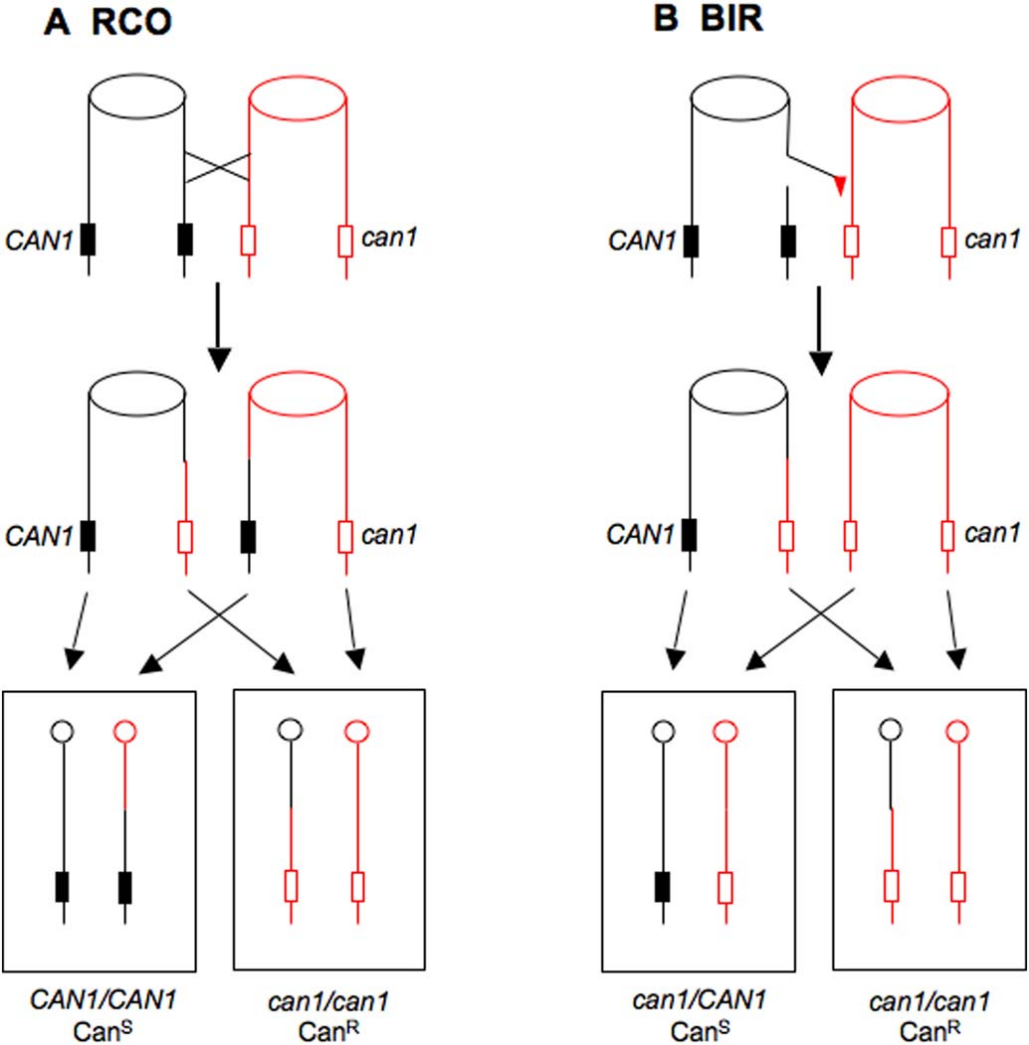
Detection of mitotic recombination events in a diploid heterozygous for the *can1* gene. The two homologues are depicted in G2 with the duplicated chromatids held together at the centromere (shown as ovals). A) Following a reciprocal crossover (RCO), one daughter cell is homozygous for the recessive *can1* allele and is canavanine resistant, whereas the other daughter cell is homozygous for the wild-type allele and is canavanine sensitive. Note that only one of the two possible chromosome disjunction patterns is shown; the other pattern does not lead to the markers becoming homozygous. B) Break-induced replication (BIR) is a fundamentally non-reciprocal process. In this depiction, the black chromatid is broken and the broken end invades the red chromatid, duplicating all the sequences to the end of the chromatid. The net result of this process is one Can^R^ *can1/can1* cell and one Can^S^ *can1/CAN1* cell.

Although mitotic recombination between homologous chromosomes was first described in 1936 [1], our understanding of the mechanism of spontaneous mitotic recombination is still limited for two related reasons. First, spontaneous mitotic recombination events are very infrequent compared to meiotic exchanges. In *S. cerevisiae*, mitotic crossovers and conversions are about 10^4^ to 10^5^-fold less frequent than meiotic events [2],[3] and usually require a selective system for their detection. Second, these systems, in general, do not allow selection of both daughter cells that contain the recombinant chromosomes generated in the mother cell. Reciprocal crossovers (RCOs) between homologous chromosomes that have a heterozygous marker can lead to daughter cells that are homozygous for the marker (loss of heterozygosity, LOH). One selective system in *S. cerevisiae* to detect such events uses the heterozygous drug-resistance marker *can1* (Figure 1A). Since diploids heterozygous for this marker are sensitive to the arginine analogue canavanine, a derivative that is homozygous for the mutant allele arising from crossing over can be selected on medium containing canavanine. The daughter cell homozygous for the wild-type *CAN1* allele, however, cannot be selected.

A canavanine-resistant diploid can also be derived from a heterozygous diploid by break-induced DNA replication (BIR) [4]. As shown in Figure 1B, a double-strand DNA break (DSB) on the *CAN1*-containing chromosome is repaired by copying the DNA from the *can1*-containing chromosome. Since the only selectable daughter cell in this system is identical for both RCO and BIR, these two mechanisms cannot be distinguished by this system. Two recent studies have examined the relative contributions of RCO and BIR to LOH in yeast. Using a non-selective approach, McMurray and Gottschling [5] showed that most LOH events in “young” cells (cells that have not undergone many mitotic divisions) represent RCOs, whereas LOH events in “old” cells often involve BIR. Using a selective approach that will be described further below, we found that most spontaneous LOH events are RCOs and recombination events induced by hydroxyurea are both RCO and BIR [6].

**Figure 2:**
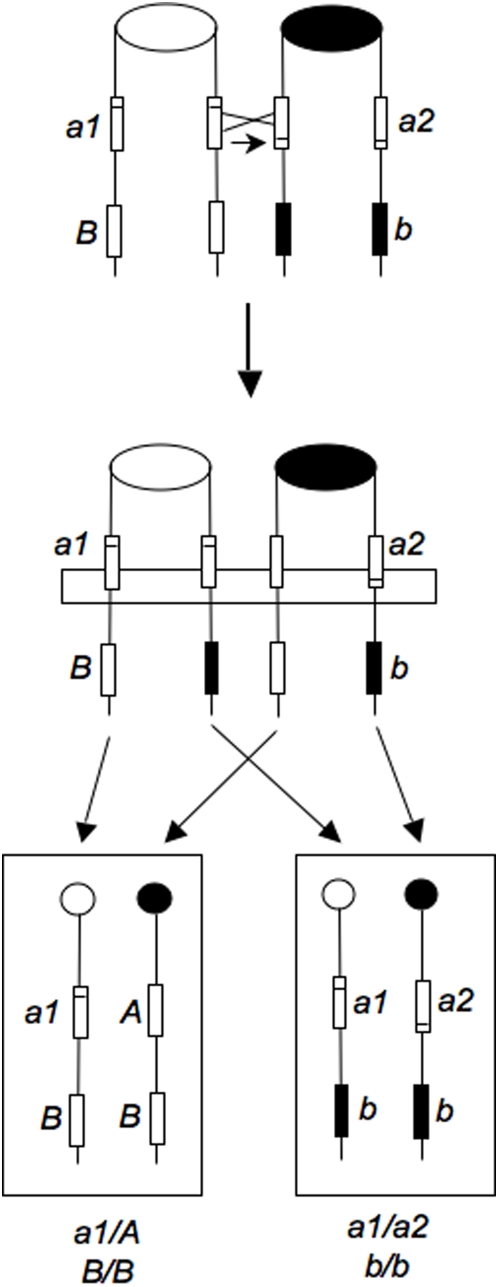
Intragenic mitotic gene conversion associated with crossing over. The two heteroalleles (*a1* and *a2*) are shown as rectangles with the position of the mutation indicated by a horizontal line within the rectangle. In this diagram, wild-type genetic information is transferred (indicated by a short horizontal arrow) from the centromere-distal part of the *a1* allele to the centromere-distal part of the *a2* allele, resulting in a wild-type *A* gene. The horizontal rectangle shows the region of gene conversion (three of the chromatids having wild-type sequences at the distal end of the gene and one having mutant sequences). The wild-type and mutant alleles of the centromere-distal marker are shown as white and black rectangles, respectively.

In mitosis, as in meiosis, gene conversion events are observed and these events are often associated with crossovers [3]. Conversion events are the non-reciprocal transfer of information between homologous DNA sequences and, in meiosis, most conversions reflect heteroduplex formation, followed by mismatch repair [7]. Most studies of mitotic conversion employ strains that are heteroallelic for an auxotrophic marker and heterozygous for a centromere-distal marker (Figure 2). Although a reciprocal crossover between the heteroalleles could produce a prototroph, Roman [8] showed that most prototrophs were a consequence of a gene conversion event. It should be noted that use of heteroalleles for the detection of gene conversion is rather restrictive. If gene conversion is a consequence of heteroduplex formation followed by mismatch repair, in order to obtain a wild-type allele by conversion, the heteroduplex must include only one of the two alleles or the repair of the heteroduplex containing both alleles must be “patchy”. As described below, we found that the mitotic conversion tracts associated with RCO in our system are usually very long and continuous.

In numerous studies of the type diagrammed in Figure 2, heteroallelic gene conversion is associated with LOH of a centromere-distal heterozygous marker. The degree of association varies between about 10% and 50% [2]. Based on the expected patterns of segregation following an RCO, one would expect that only half of the RCOs would be detectable by producing cells that have undergone LOH (Figures 1 and 2). Chua and Jinks-Robertson [9] showed that this expectation is met for *S. cerevisiae*, although in Drosophila, the crossover chromatids usually segregate into different daughter cells [10].

Stern [1] argued that mitotic crossovers occur in G2 (as shown in Figures 1 and 2) because a mitotic crossover between unreplicated chromosomes would not result in LOH for heterozygous markers (assuming that the chromosomes undergo an equational division). In *S. cerevisiae*, however, two studies demonstrated that mitotic gene conversion could be induced in G1 cells by ultraviolet light or gamma rays [11],[12]. From his analysis of crossovers associated with heteroallelic gene conversion events, Esposito [13] suggested that spontaneous mitotic exchanges also occur in G1. He argued that Holliday junction intermediates formed in G1 were replicated rather than resolved by junction-cleaving enzymes, generating G2-like crossovers. In the analysis described below, we present evidence that at least 40% of spontaneous RCOs are initiated in G1.

## Results

### Experimental Rationale

**Figure 3:**
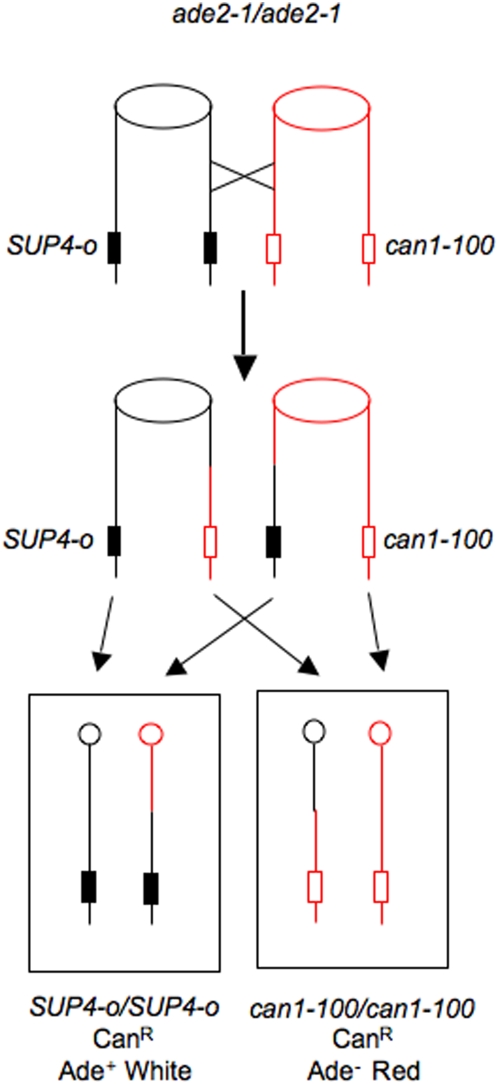
A diploid strain that allows the selection of both products of an RCO. The *SUP4-o* gene encodes a tRNA that suppresses both the *can1-100* and *ade2-1* alleles. Strains that have these mutations in the absence of the suppressor are canavanine resistant, adenine auxotrophs, and form red colonies (because of the accumulation of a pigmented precursor to adenine). In the presence of the suppressor, the strains are canavanine sensitive, adenine prototrophs, and form white colonies. If there is an RCO between the centromere and the *can1-100/SUP4-o* markers, two Can^R^ cells will be produced; subsequent divisions of these cells will result in a red/white Can^R^ sectored colony.

We previously described a genetic system (Figure 3) allowing for the selection of both daughter cells containing the reciprocal products of mitotic crossovers in the 120 kb *CEN5-CAN1* interval on chromosome V [6]. One homologue has the *can1-100* allele, an ochre mutation. On the other homologue, the *CAN1* gene has been replaced with *SUP4-o*, a gene encoding an ochre-suppressing tRNA [6]. In addition, the diploid is homozygous for *ade2-1*, also an ochre mutation. In the absence of a suppressor, strains with the *ade2-1* mutation require adenine, form red colonies because of the accumulation of a red precursor to adenine, and are canavanine-resistant. The starting diploid strain is canavanine-sensitive (Can^S^) and forms white colonies. If an RCO occurs between *CEN5* and the *can1-100/SUP4-o* markers as the cells are plated on canavanine, a red/white sectored Can^R^ colony will be formed.

In our first use of this system, the two homologues were derived from isogenic haploids, resulting in a diploid that had no polymorphisms. In the current study, using standard methods [14], we constructed a diploid by crossing the haploid strains W303A and YJM789. These strains have about 0.5% sequence divergence and, therefore, about 60,000 single-nucleotide differences [15]; S288c and W303A are closely related in sequence [16]. By comparisons of the genomic sequences, we identified 34 polymorphisms between W303A and YJM789 in the *CEN5-CAN1* interval and used those polymorphisms to map crossovers and associated gene conversion tracts as described below. The diploids derived from crossing W303a- and YJM789-derived strains were PSL100 and PSL101. These strains are identical except one strain (PSL100) is homozygous for the *ura3* mutation and the other (PSL101) is heterozygous *ura3/URA3*; these strains yielded very similar results.

Each red/white sectored Can^R^ colony reflects an independent RCO (Figure 3). We isolated genomic DNA from cells purified from the red and white sectors and analyzed the segregation of the polymorphisms by PCR followed by restriction enzyme treatment (details in Materials and Methods). For example, one polymorphism distinguishing W303A and YJM789 is located at SGD coordinate 60,163 on chromosome V. A *Hpy188*III site that is present at this position in the W303A genome is absent in the YJM789 genome. We designed primers flanking this site (Table S2) that result in a PCR product of about 520 bp. Thus, if we amplify genomic DNA from a diploid that is homozygous for the W303A form of the polymorphism, treat the amplified product with *Hyp188*III, and analyze the products by agarose gel electrophoresis, we observe two fragments of about 250 and 270 bp. A strain homozygous for the YJM789 form of the polymorphism produces a single fragment of 520 bp, and a heterozygous diploid produces three fragments of 250, 270, and 520 bp.

**Figure 4:**
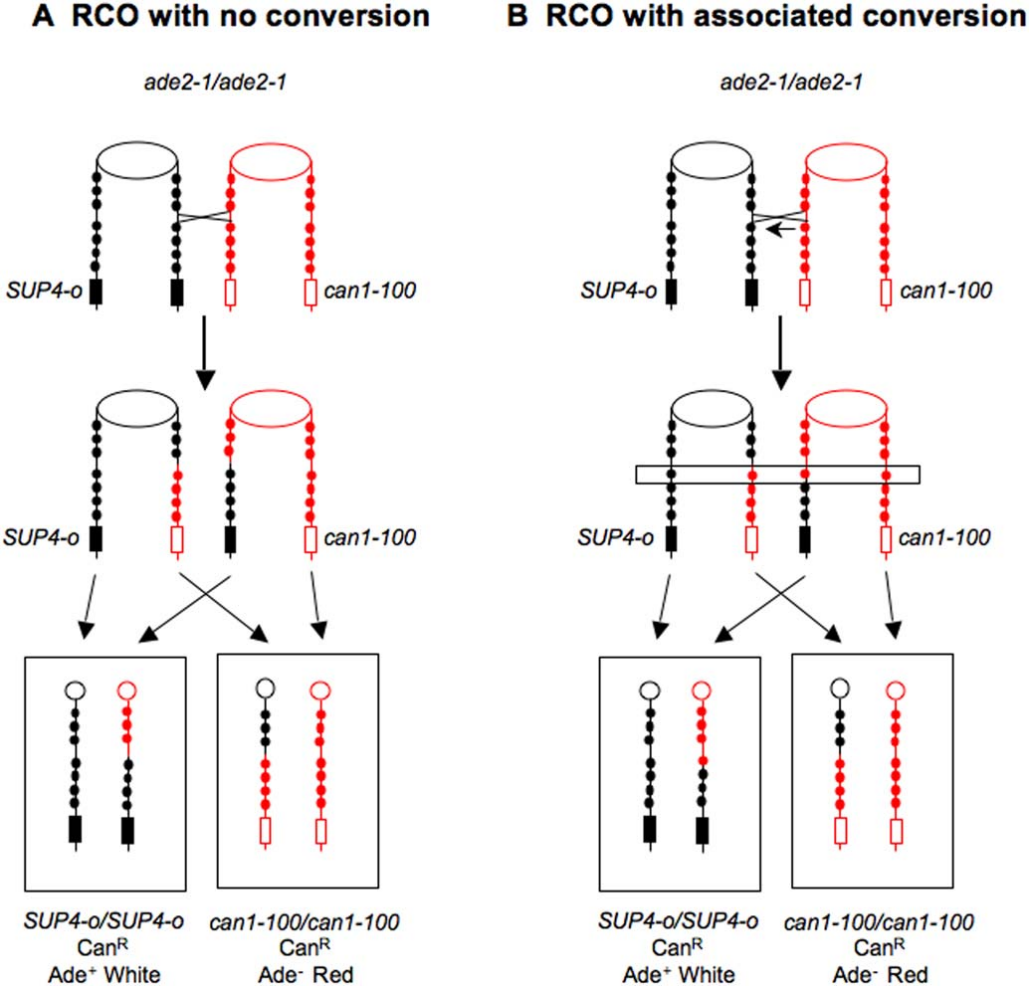
Segregation patterns of heterozygous markers after RCO. In this figure, the heterozygous markers are depicted as circles. Only seven of the 34 heterozygous markers in the *CEN5-CAN1* interval are shown. The red and black colors represent markers derived from the W303A- and YJM789-related chromosomes, respectively. A) Following an RCO that had no associated conversion, both sectors are heterozygous for all markers centromere-proximal to the exchange. Distal to the exchange, the white sector is homozygous for the YJM789 markers and the red sector is homozygous for the W303A markers. B) This diagram shows a conversion event (indicated by the arrow) in which one of the black markers is lost and one of the red markers is duplicated. For this marker (boxed with the horizontal rectangle), three of the chromatids have the red marker and one has the black marker. Proximal to the conversion and associated crossover, the markers in both sectors are heterozygous; distal to the conversion/crossover boundary, the markers are homozygous with the same patterns observed in Figure 4A.

The patterns of marker segregation that were expected are shown in Figure 4. For a RCO unassociated with gene conversion (Figure 4A), we expect that markers centromere-proximal to the exchange will be heterozygous in both the red and white sectors. Centromere-distal to the exchange the sectors should be homozygous, the red sector homozygous for the W303A markers and the white sector homozygous for the YJM789 markers. If there is a conversion associated with the RCO (Figure 4B), there will also be a region in which a marker is heterozygous in one sector but homozygous in the other. Such a segregation pattern is analogous to a 3∶1 meiotic segregation event.

### Rates of RCOs in PSL100/101 and Related Strains

Before mapping the crossovers and associated conversion events, we determined the rate of RCOs. In our previous study with a diploid (MAB6) that was constructed from a cross of two W303A-related haploids and had no polymorphisms between *CEN5* and *CAN1* [6], we observed Can^R^ red/white sectors at a rate of 2±0.6×10^−5^/division (±95% confidence limits); this analysis was done in cells cultured at 30°C. Since the background growth of Can^S^ cells on the canavanine-containing solid medium in the W303A/YJM789 diploid used in the present study is strong at 30°, we performed all experiments at 22°. At this temperature, the rate of Can^R^ red/white sectors in MAB6 was reduced to 2.9±0.4×10^−6^/division. The rate of Can^R^ red/white sectors in PSL101 (the diploid with the hybrid W303A/YJM789 background) was 3.3±0.2×10^−6^/division, indicating that the numerous sequence polymorphisms do not significantly affect the rate of RCOs. Since only half of the segregation events in cells with an RCO result in loss of heterozygosity [9], the calculated rate of RCO in PSL101 (about 7×10^−6^) is twice the rate of sector formation.

We also examined the rates of Can^R^ red/white sectors in PG311 and MD457, *MATa/MATαΔ* and *spo11/spo11* derivatives of PSL101, respectively. The rates of sectors were 1.1±0.5×10^−6^/division (PG311) and 0.8±0.1×10^−6^/division (MD457). Since we previously found no significant effect of heterozygosity at the *MAT* locus on RCOs [6] and since Spo11p is not expressed in vegetative cells [17], the significance of the three-fold reduction in the rate of RCOs relative to PSL101 is unclear. As will be described below, the patterns of segregation of polymorphisms in MD457 and PG311 were very similar to those observed in PSL101.

### Mapping of Mitotic Crossovers and Gene Conversions in PSL100 and PSL101

**Figure 5:**
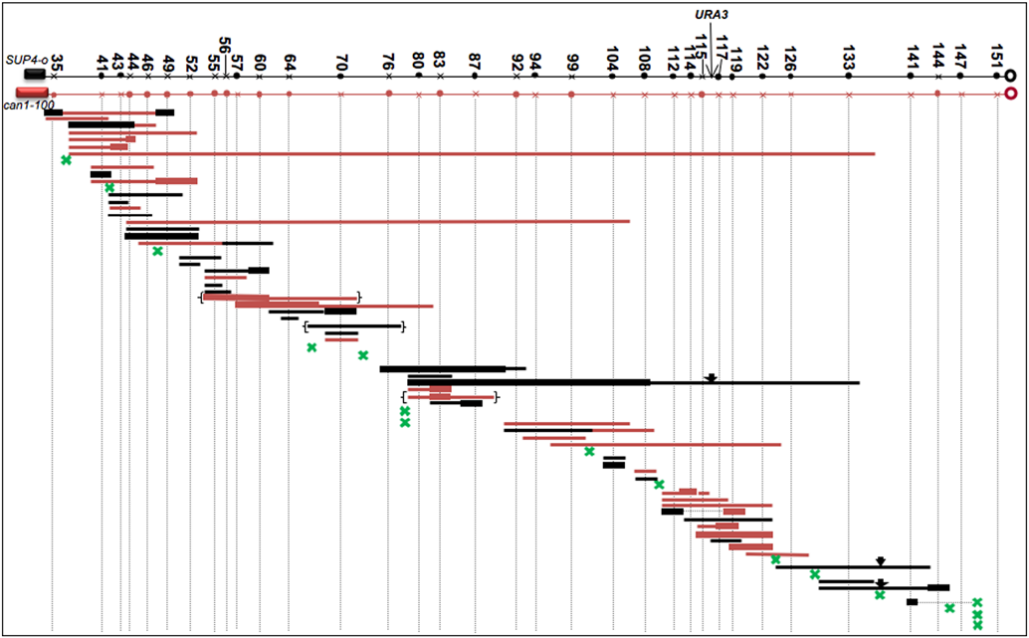
Mapping of RCOs and associated gene conversion tracts in the *CEN5-CAN1* interval. Thirty-four markers were used to map events in 74 independent red/white Can^R^ colonies; both sectors were analyzed by methods described in the text. The positions of the markers are shown by circles and X's on the two chromosomes, with the circle indicating that the diagnostic restriction site exists and the X indicating that the site does not exist. The numbers associated with the markers represent approximate SGD coordinates in kb. *CEN5* is located at about SGD coordinate 152,000, and *CAN1* is located at about position 33,000. Green X's show the positions of RCOs that are not associated with a gene conversion tract. Thin horizontal lines show the extent of “normal” 3∶1 gene conversion tracts and thick lines show 4∶0 conversions. The color indicates whether the markers donated in the conversion event were derived from the homologue with the YJM789 (black) or W303A (red) markers. For example, a thin red line indicates that one sector was homozygous for the markers derived from W303A and the other sector was heterozygous for these markers. For most of the conversion tracts, the crossover maps adjacent to the tract. For those tracts with an arrow above the tract, the crossover occurred within the conversion tract. The tracts in brackets have markers in the unexpected association as discussed in the text. In addition, for two of the tracts, the position of the crossover was separated from the conversion tract; these events are shown with a dotted line connecting the tract and the crossover.

We mapped crossovers and conversions in 74 Can^R^ red/white sectored colonies derived from PSL100 and PSL101. The locations of the mapped events are shown in Figure 5. Green Xs indicate crossovers unassociated with gene conversion and the horizontal lines indicate the extent of gene conversion tracts associated with crossovers (red and black lines indicating markers derived from the W303A- and YJM789-derived homologues, respectively). Several generalizations can be made based on our analysis. First, most (59 of 74; about 80%) of the RCOs are associated with adjacent conversion tracts; the conversion tract is adjacent to the crossover in 58 of the 59 tracts. For most conversion events (exceptions to be discussed below), we cannot determine whether the crossover occurred within the tract or at one of the two ends of the tract. Second, most (54 of 59) of the tracts are exclusively red or exclusively black, indicating that only one homologue was the donor in each conversion event. Third, the red and black conversion tracts are not usually interrupted by markers that do not undergo conversion, demonstrating that regions of DNA from one homologue are usually non-reciprocally transferred as a single entity to the other homologue. Fourth, since the numbers of red and black conversion tracts (26 and 28, respectively) are approximately equal, the two homologues are equally capable of donating information during a conversion event. Fifth, although about 20% of the crossovers have no detectable conversion tracts, it is likely that most or all of such crossovers are associated with conversion events that could be detected with a denser array of markers.

In addition to the “normal” (3∶1) gene conversion events shown as thin lines in Figure 5, we detected an unexpected type of conversion. In this class (which we term “4∶0” conversion), the same form of the polymorphism was homozygous in the red and white sectors. These conversion tracts are shown as thick lines in Figure 5. Of the 59 conversion events observed, 35 were 3∶1 conversions, 7 were 4∶0 conversions, and 17 were hybrid 3∶1, 4∶0 tracts. The 4∶0 tracts and hybrid tracts are unlikely to reflect two independent events, since the frequency of these tracts is similar to that observed for the 3∶1 tracts. In addition, about 70% of the 4∶0 tracts are contiguous with a 3∶1 tract and, in 15 of the 17 hybrid tracts, the 4∶0 segment of the tract is derived from the same chromosome as the 3∶1 tract (Figure 5). Our favored interpretation of the 4∶0 conversion events (outlined in detail in the Discussion) is that they are a consequence of a double repair event of a chromosome that was broken in G1 and replicated to yield two broken chromatids.

There were 48 3∶1 or hybrid conversion tracts that involved sequences donated exclusively from W303A or YJM789. In Figure 4B, we show the red chromatid (representing W303A sequences) donating sequences to the black chromatid during the conversion event. For this type of event, we expect that the red sector (homozygous for *can1-100*) will be homozygous for the converted marker(s) and the white sector (homozygous for *SUP4-o*) will be heterozygous for these marker(s). This expected pattern was observed in 42 of the 48 conversion events with a 3∶1 or hybrid 3∶1/4∶0 tract. In three of the 48 events, the patterns of markers in the sector were in the opposite direction (defined as the “unexpected” pattern) and, in three events, the patterns suggested a crossover within the 3∶1 conversion tract. These unusual patterns of marker segregation may reflect repair of a G1-associated DSB and are discussed further in the Supporting Information (Text S1, Figures S1 and S2). For both meiotic and induced mitotic gene conversion events, the chromosome with the DNA lesion that initiates the exchange (for example, a double-strand break) is the recipient of genetic information [3]. Our data do not address this issue for spontaneous mitotic events.

The analysis described above can determine whether the strain is heterozygous or homozygous for markers but does not reveal the coupling of heterozygous markers. Our expectation was that in sectors with heterozygous markers, the original coupling of these markers was maintained, one chromosome containing the W303A-derived markers and the other the YJM789-derived markers. This expectation was checked for the red and white sectors of nine sectored colonies. Strains derived from each sector were sporulated and we analyzed the segregation of multiple heterozygous markers in the four spores. For the heterozygous markers, we found that two of the spores had markers derived from W303A and two had markers from YJM789, indicating that heterozygous markers usually had the same coupling relationship as in the chromosomes before the mitotic exchange.

We classified 47 of the 59 conversion tracts in our study as “simple” using the following criteria: 1) the tract is continuous and the converted sequences are derived from only one of the two homologues, 2) the crossover is adjacent to the conversion tract, and 3) the 3∶1 conversion tract has the expected association (as defined above) with the sector. We included 3∶1, 4∶0, and hybrid tracts in our analysis. Most of these tracts spanned more than one marker. For each conversion event, we estimated the tract size by averaging the maximum tract size (the distance between markers that flanked the conversion tract) and the minimum tract size (the distance between markers that were included within the tract); for conversion events that included one site, the minimum tract size was taken to be one bp. The tract size averaged for the 47 events was 11.7±1.6 kb (95% confidence limits); the median track size was 7.6 kb. We also calculated the average tract lengths separately for 3∶1 events (12.6±2.4 kb), 4∶0 events (6.8±0.8 kb), and hybrid events (11.4±1.2 kb). These tracts are considerably longer than those observed in meiotic cells that average about 1–4 kb [18]–[21]. The sizes of all conversion tracts for PSL100/PSL101 and the other strains used in this study are in tables in the Supporting Information section (Tables S3, S4, S5, and S6).

As discussed above, the mitotic crossovers that had no detectable conversion event are likely to have had a conversion tract that was restricted to the region between the assayed markers. If we assume that these postulated conversion events had tract sizes that were half of the distance between the markers in the interval containing the crossover, then the average mitotic conversion tract for PSL100/PSL101 was 9.4 kb rather than 11.7 kb, still considerably longer than meiotic conversion tracts estimated in other studies. In summary, our analysis of mitotic crossovers indicated two unusual features of the gene conversion tracts associated with the RCO: the tracts were often very long, and about 40% of the tracts were not consistent with the simplest model of a G2-initiated recombination event.

### Mapping of Mitotic Crossovers and Gene Conversions in MD457 and PG311

**Figure 6:**
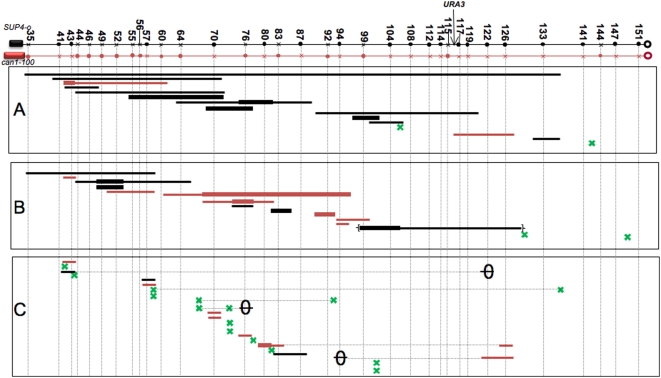
Mapping of mitotic crossovers in meiosis-deficient derivatives of PSL101 (MD457 and PG311) and meiotic crossovers and conversions in PSL101. The depictions of crossovers and conversions are the same as in Figure 5. A) Analysis of crossovers and conversions in 14 sectored colonies derived from MD457, an isogenic *spo11/spo11* derivative of PSL101. B) Analysis of crossovers and conversions in 15 sectored colonies derived from PG311, an isogenic *MATa/MATαΔ::NAT* derivative of PSL101. C) Meiotic crossovers and conversion in PSL101. The diploid was sporulated and the segregation of markers in the spores was examined. Conversion tracts that were unassociated with crossovers are indicated by a horizontal line with a superimposed oval. Multiple events within one tetrad are shown with a connecting dotted line. Two conversion events that include the *can1-100/SUP4-o* marker are not shown.

To ensure that the unusual gene conversion events described above were not a consequence of a sub-set of cells that underwent meiotic levels of recombination, followed by mitotic patterns of chromosome disjunction, we examined mitotic recombination in MD457 (a *spo11/spo11* derivative of PSL101) and PG311 (a *MATa/MATαΔ* derivative of PSL101). These strains are incapable of meiotic recombination. The positions of RCOs and their associated conversion tracts (14 from MD457 and 15 from PG311) are shown in Figure 6. The types of conversion events are similar to each other and to those observed in PSL100/PSL101. The gene conversion tracts were very long in the two strains, and we observed 3∶1, 4∶0, and hybrid 3∶1/4∶0 tracts in approximately the same proportions as in PSL101. The average conversion tract sizes (average of all three types) were 26.2±5.1 kb for MD457 and 12.8±2.3 kb for PG311; the median track sizes for MD457 and PG311 were 20.1 kb and 6.1 kb, respectively. The average conversion tract size for MD457 is somewhat misleading because one very large tract (103 kb) had a substantial effect on the average. The average tract size for the other tracts in MD457 was 19.2 kb. These results argue that the very long conversion tracts and 4∶0 and 3∶1/4∶0 classes of events observed in PSL101 do not reflect an aberrant type of meiotic recombination.

### Meiotic Crossovers and Associated Gene Conversions

Using methods similar to those used to map mitotic crossovers and conversions, we also examined the patterns of meiotic exchanges in 21 tetrads derived from PS101. By examining the segregation of the centromere-linked *trp1* marker and the *can1-100/SUP4-o* markers, we identified tetrads that had at least one crossover in the 120 kb *CEN5-CAN1* interval. The positions of crossovers and the lengths of associated gene conversion tracts in these tetrads are shown in Figure 6C. Eleven of the conversion tracts were associated with crossovers and three were not. Of the eleven tracts associated with crossovers, eight included only one marker and three included two. None of the conversion sites spanned more than two markers. In striking contrast, of the 47 “simple” conversion events associated with mitotic crossovers in the same strain, as described above, 12 included only one marker, 12 spanned two markers, and 23 involved more than two markers. This difference in the sizes of meiotic and mitotic tracts is very significant (p value of 0.001 by Fisher exact test). In addition, using the same methods to estimate conversion tract length that we used for mitotic tracts, we calculated the average meiotic conversion tract length in PSL101 as 4.7±0.6 kb, significantly (p<0.05) less than that observed in mitosis. If we assume that the crossovers with no detectable conversions had tracts that were half of the size of the interval between the markers containing the crossovers, the average conversion tract was 3.2 kb. In summary, these results demonstrate that the long mitotic conversion tracts in PSL101 and related strains are not an artifact generated by the high level of polymorphisms in PSL101 and related diploids, but reflect differences in the mechanisms of meiotic and mitotic recombination.

As expected from many previous studies [3],[7], most of the meiotic conversion events are 3∶1 events (three spores with one form of the polymorphism, one with the alternative form), but one tetrad had a conversion tract with a “4∶0” segment adjacent to a 3∶1 segment, similar to some of the mitotic conversion tracts described previously. Meiotic conversion events with 4∶0 segregation have been seen previously at meiotic recombination hotspots [22] and occur at the frequency expected for two independent conversion events.

In 21 tetrads, we observed 26 crossovers; about 40% (11) were associated with conversion tracts and 60% (15) were not. This association between meiotic crossovers and conversion is significantly less (p value≤0.001 by Fisher exact test) than observed for mitotic crossovers and conversion in PSL100/101 where 59 of 74 crossovers were associated with a conversion tract. A simple interpretation of this result is that the longer conversion tracts associated with mitotic crossovers make it more likely that an associated conversion event will be detectable in mitotic cells than in meiotic cells.

## Discussion

In this study, we show that most spontaneous reciprocal crossovers are associated with long gene conversion tracts. In addition, we found that about 40% of the conversion tracts had an unusual pattern in which one form of the polymorphism became homozygous in both sectors (4∶0 conversion); as described below, we interpret such conversion tracts as representing the repair of a G1-initiated DNA lesion. Below, we discuss: 1) the distribution of mitotic gene conversion events in the *CEN5-CAN1* interval, 2) a comparison of the lengths of mitotic and meiotic conversion tracts, and 3) mechanisms of mitotic recombination.

### Distribution of Mitotic Recombination Events

Meiotic recombination events in *S. cerevisiae* are distributed non-randomly. Certain chromosomal domains have low levels of exchange (for example, near the centromeres and telomeres) and there are intergenic regions with very elevated rates of recombination (hotspots) correlated with high levels of local meiosis-specific double-strand DNA breaks [23],[24]. Although no high-resolution mitotic recombination maps have been constructed previously, several DNA sequence motifs or conditions have been associated with elevated rates of mitotic recombination in yeast including: elevated rates of transcription, replication fork pausing/stalling, and DNA sequences capable of forming secondary structures such as poly CCG or inverted repeats [25]. Most of the assays of the recombination-stimulating sequences involve recombination between direct or inverted repeats rather than recombination between homologous chromosomes.

**Figure 7:**
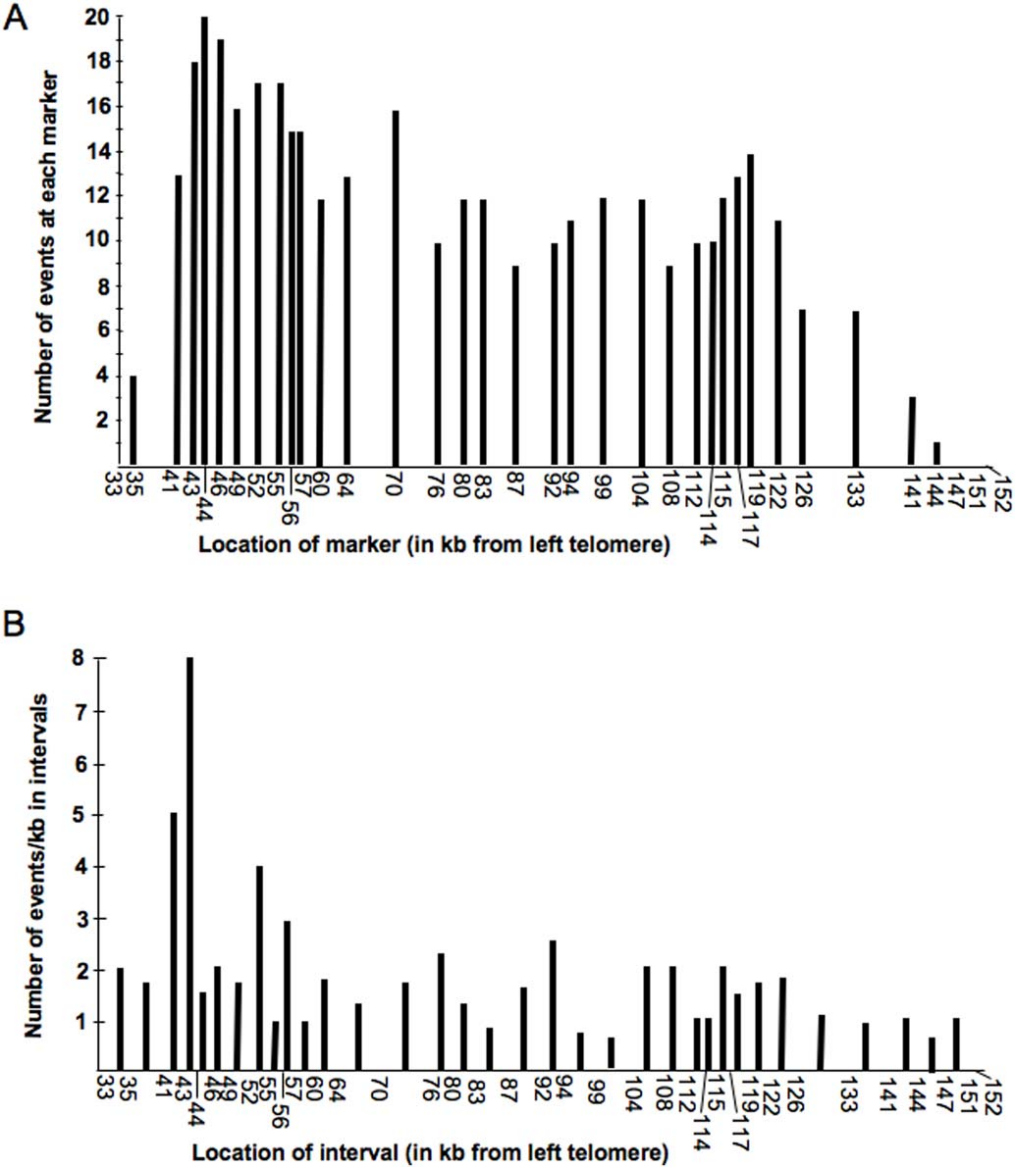
Distribution of mitotic recombination events in the *CEN5-CAN1* interval. This figure is a summary of the distribution of mitotic recombination events in the strains PSL100, PS101, MD457, and PG311. A) For each marker, we summed the conversion events that include the marker over all of the strains. Both simple and complex conversion events were used in this analysis. B) For each interval, we summed the conversion tracts that end in the interval and the crossovers within the interval. We then divided that sum by the length of the interval in kb.

From the patterns of the spontaneous recombination events shown in Figures 5 and 6, it is clear that crossovers and conversions are initiated at many sites within the *CEN5-CAN1* interval, although there appear to be more conversion tracts near *CAN1* than near the centromere. This impression is conveyed more clearly in Figure 7A. In this figure, we show the number of times each marker was involved in a conversion event in the strains PSL100/101, MD457, and PG311. If we divide the region into four intervals of approximately the same size and sum the number of events/marker over all markers in each quadrant, we find 124 (Quadrant 1, markers 35 to 55), 112 (Quadrant 2, markers 56–87), 99 (Quadrant 3, markers 92–117), and 43 (Quadrant 4, markers 119–151) events in each quandrant, moving from *CAN1* to *CEN5*. This distribution of events is very significantly different (p = <0.0001 by chi-square test) from random. In addition, the number of events in Intervals 1 and 4 are significantly greater and less, respectively, than that expected from a random distribution.

We confirmed this conclusion using two other types of analysis. First, we determined the number of conversion tracts within each quadrant. Only tracts that did not span two different quadrants were included. We found 28, 16, 18, and 4 tracts within the Quadrants 1–4, respectively. This distribution was significantly different from random (p = 0.0005). One difficulty in localizing a mitotic recombination hotspot is that the conversion tracts are long and heterogeneous in length. In meiosis, although the initiating DNA lesion stimulates gene conversion tracts bidirectionally, individual gene conversion tracts are propagated unidirectionally from the initiating DNA lesion [26]. In an analysis of HO-induced mitotic gene conversion tracts [27], about 80% of the tracts were bidirectional from the DSB site, although the length of DNA transferred was often much greater on one side of the DSB site than the other. If we assume that individual spontaneous conversion events are propagated predominately in a single direction from the initiating lesion, one of the endpoints of the conversion tract will be near the initiating DNA lesion. Thus, we determined the number of conversion tracts that ended in each of the 35 intervals defined by the polymorphic markers; we also included in this analysis the crossovers within each interval. When these events were summed within each quadrant, we found 65, 54, 38, and 31 events, respectively, in Intervals 1–4. This distribution of events is significantly (p = 0.0006) different from random. In Figure 7B, we show the number of events (termini of conversion tracts and crossovers) within each of the 35 intervals, normalized for the size of the interval. A peak between markers 43 (SGD coordinates 43078) and 44 (SGD coordinates 44403) is evident. The observed number of events (8) in this 1.3 kb interval is significantly (p<0.0001 by chi-square analysis) in excess of that expected based on a random distribution of 188 events in the 119 kb *CAN1-CEN5* interval.

The interval between markers 43 and 44 includes part of the *PCM1* gene and the *SOM1-PCM1* intergenic region. As discussed above, elevated levels of mitotic recombination have been associated with certain types of DNA structures (inverted repeats), microsatellite sequences, or high levels of transcription. There are no obvious structure/sequence elements in the 1.3 kb region, and *SOM1* and *PCM1* are not among the most abundant transcripts in the yeast genome [28]. We also compared the level of mitotic recombination for each marker (measured as in Figure 7A) with the level of gene expression of the ORF closest to the marker [28] by a linear regression analysis; no significant correlation was observed (r^2^ = 0.004; p = 0.74). An understanding of the nature of mitotic recombination hotspots will probably require identification and analysis of many hotspots.

Several other points should be made concerning the distribution of mitotic events. First, the frequency of gene conversion events near the *CAN1* gene is somewhat underestimated, since a conversion event extending through the *can1-100/SUP4-o* markers would not result in a Can^R^ red/white sectored colony. Second, in our previous study of mitotic recombination [6], we did not observe a reduction of exchange in the 35 kb *URA3-CEN5* interval. In this previous study, however, our estimate of crossovers was based on a relatively small number of events and was insensitive to a small degree of suppression. From our current study, it is possible that mitotic recombination, like meiotic recombination, is reduced close (within 20 kb) to the centromere. This conclusion, however, is tentative until studies of mitotic recombination have been extended to multiple chromosomes. In addition, although mitotic recombination is reduced near *CEN5*, gene conversion events can extend through the centromere [29]. In summary, our analysis of the distribution of mitotic recombination events demonstrates that these events can be initiated at many locations in the *CAN1-CEN5* interval, although we have preliminary evidence of one mitotic recombination hotspot.

By a variety of microarray-based procedures, we and others have measured the distribution of meiosis-specific DSBs throughout the yeast genome [30]–[34]. We compared the number of mitotic conversion events involving each polymorphic site (Figure 7) with the meiotic recombination activity of the nearest ORF (derived from Table S2) [32] by a linear correlation and regression analysis. No significant correlation was observed (r^2^ = 0.021; p = 0.41 by two-tailed test). Since meiotic recombinogenic lesions are generated by Spo11p which is not expressed in mitotic cells, this result is not unexpected.

### A Comparison of the Lengths of Mitotic and Meiotic Conversion Tracts

Before comparing mitotic and meiotic conversion events, we will briefly compare previous studies of mitotic conversions in yeast with our study. In our study, only mitotic conversion tracts associated with crossovers were examined. In a number of studies [35], it was shown that mitotic conversion tracts associated with crossovers are longer than conversion tracts unassociated with crossovers. Most previous studies of mitotic conversion and crossovers were done using systems in which the length of the conversion was constrained in one of two ways. First, in studies involving inverted or direct repeats, the sizes of the conversion tracts are limited by the size of the repeats. Second, in experiments involving selection of a prototroph from a heteroallelic diploid, the system is biased against long continuous conversion tracts, the type of tract that is most common in our study.

Nickoloff *et al.* [27] analyzed gene conversion events between homologous chromosomes in which an HO-induced DSB within the *URA3* gene was the initiating lesion. The diploid strain was also heterozygous for markers flanking the HO cleavage site, approximately two kb to one side and 1 kb to the other. Most of the tracts were continuous, and 60% extended outside of the markers on one side or the other; 30% were beyond all of the markers, a minimal distance of 3.4 kb. In an analysis of 51 spontaneous mitotic conversion events *unassociated* with crossovers, Judd and Petes [19] found 49 that were greater than two kb, and 19 of these 49 were greater than four kb (end points extending beyond the markers). 50 of the 51 tracts in this study were continuous. Using a different approach, Golin and Esposito [36] examined co-conversion of heteroalleles located about 30 kb apart on chromosome VII. Although the rate of co-conversion events was 50-fold less than the rates of conversion at one locus or the other, these co-events were 1000-fold more frequent than expected for independent events, arguing the possibility of rare very long mitotic conversion tracts. Although very long conversion tracts could reflect BIR [4], co-conversion of two pairs of heteroalleles is unlikely to be a consequence of BIR.

With the exception of the current study, there is only one analysis of meiotic and mitotic conversion events in the same genomic region of the same strain [19]. Of ten meiotic conversion tracts, eight had two defined endpoints (compared to 11 of 51 mitotic events). The average size of these eight tracts was 2.1 kb, clearly shorter than the mitotic tracts. In two other meiotic studies using similar methods, average conversion tract lengths of 3.4 kb [18] and 1.5 kb [20] were observed. Because Borts and Haber [20] calculated the minimal tract lengths rather than the average of the minimal and maximal lengths, these two estimates are not significantly different.

The most accurate estimates of meiotic conversion tracts can be obtained in strains with the maximum density of markers with the caveat that the markers themselves could influence the pattern of gene conversion [37]. In a genetic background very similar to one used in our study, Mancera *et al.* [21] used high-density microarrays to map meiotic crossovers and gene conversions with markers that had a median spacing of about 80 bp. In analyzing several thousand conversion events, Mancera *et al.* found an average tract length of 2.0 kb for conversions associated with crossovers and 1.8 kb for conversions unassociated with crossovers. In summary, our analysis, as well as those of others, demonstrates that meiotic conversion tracts are considerably shorter than mitotic conversion tracts.

### Mechanisms of Mitotic Recombination

We will discuss three related aspects of the mechanism of mitotic recombination: 1) the timing of the initiating DNA lesion in the cell cycle, 2) the nature of the initiating DNA lesion, and 3) mechanisms of generating long continuous mitotic conversion tracts.

#### Timing of the initiating DNA lesion in the cell cycle

**Figure 8:**
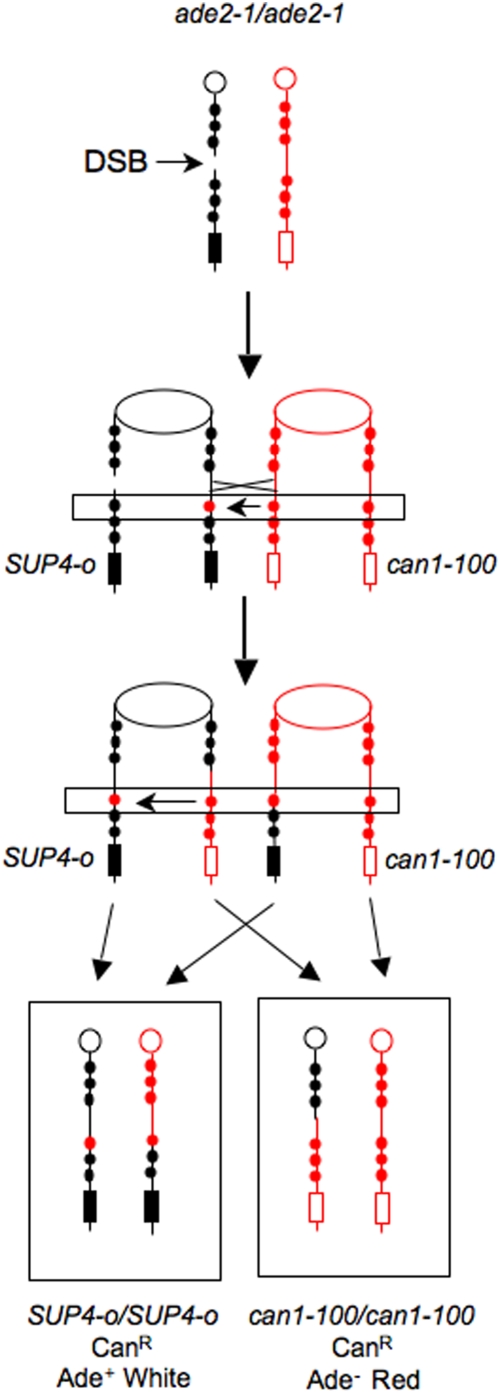
Mechanism to generate a 4∶0 conversion event. The recombination event initiates by a DSB in G1 on the black chromosome. The broken chromosome is replicated to yield two broken black chromatids. In gene conversion events initiated by a DSB, the broken chromatid is the recipient of information [3]. Repair of the first broken chromosome is associated with a conversion event in which the red marker is duplicated, and there is an associated crossover. Repair of the second broken chromatid could occur by an interaction with the sister chromatid (as shown) or with one of the two non-sister chromatids. This repair event would produce a second gene conversion, resulting in the 4∶0 class of event. If the first repair event had a longer conversion tract than the second, a hybrid 4∶0/3∶1 conversion tract would be formed.

One very striking feature of our data is the high frequency (about 40%) of crossover-associated conversion tracts in which a marker derived from one homologue in the original diploid has become homozygous in both sectors (4∶0 events). Our favored model to explain these tracts is shown in Figure 8. We suggest that one of the two homologues is broken in G1, and the broken chromosome is replicated. As expected from the repair of HO-induced DSBs [3], the broken chromosome would be the recipient of information during a conversion event. In Figure 8, the first DSB is repaired using the homologue as a template and this conversion event is associated with crossing-over. The second DSB could also be repaired either by using the other homologue or the newly-repaired sister-chromatid as the template to produce the 4∶0 tract. This same mechanism would produce a hybrid 3∶1/4∶0 tract if the length of the first conversion tract is longer than that of the second. For example, if the first tract was 15 kb and the second tract was 5 kb, we would detect a hybrid tract with a 5 kb 4∶0 portion and a 10 kb 3∶1 portion. The location of the 4∶0 region in the hybrid tract would depend on whether the conversion event was unidirectional or bidirectional from the initiating DSBs.

As discussed in the Introduction, two studies showed that mitotic recombination events could be induced in G1-arrested cells by UV damage or X-rays [11],[12], although these findings are not directly relevant to the issue of the timing of spontaneous mitotic recombination events. Based on a complex genetic analysis (described in detail in the Supporting Information), Esposito [13] concluded that a substantial fraction of spontaneous mitotic recombination was initiated in G1. His model to explain these results involves formation of a single Holliday junction between unduplicated chromosomes and resolution of this junction by DNA replication rather than the action of resolvase (Figure S3). In *S. cerevisiae*, repair of meiotic DSBs is associated with two adjacent Holliday junctions [38],[39], although in *S. pombe*, crossovers result from resolution of a single Holliday junction [40]. In our view, the model shown in Figure 8 is a more plausible explanation of the data.

A number of experiments demonstrate that the repair of a DSB generated in G1 has different properties from one induced in S or G2. In haploid yeast cells, DSBs induced by the HO endonuclease in G1 have very reduced levels of resection [41]–[43] and are often repaired by non-homologous end-joining events [44]. Rad53p is not activated in response to an HO-induced DSB in G1 [45], and Rad52p is not recruited to the broken DNA ends [46]. In contrast, broken DNA ends resulting from ionizing radiation treatment of G1 haploids are resected [47], although this resection does not result in phosphorylation of Rad53p except at very high doses of radiation [48]. As observed for the HO-induced DSBs, Rad52p is not recruited to the broken DNA ends [47]. These results, taken together, suggest that DSBs formed in G1 are unlikely to be repaired by homologous recombination in G1. In addition, since non-homologous end joining of broken ends is suppressed in *MATa/MATα* strains [44],[49],[50], a chromosome with a spontaneous DSB in G1 would be likely to be replicated rather than be repaired.

It is quite likely that recombinogenic DNA lesions occur throughout the cell cycle. In our study, although we interpret the 4∶0 events and 4∶0/3∶1 hybrid events as representing G1-initiated DNA lesions, 60% of the conversion tracts had the 3∶1 pattern expected for S- or G2-initiated DNA lesions. In addition, our system was designed to detect mitotic crossovers between homologous chromosomes. Repair events between sister-chromatids, a preferred pathway for X-ray-induced DNA damage in G2 cells [51] are undetectable by our analysis. Thus, a simple interpretation of our data is that many of the recombination events involving homologues are initiated in G1, since DNA lesions occurring in G2 are usually repaired using the sister chromatid as the template. It should be emphasized that we cannot determine the relative frequency of recombinogenic lesions in various portions of the cell cycle, since we cannot assay sister-chromatid exchanges with our system.

#### Nature of the DNA lesion that initiates mitotic recombination

Because of the low rate of spontaneous mitotic recombination events, there is no direct physical evidence of the nature of the recombinogenic lesion. As described above, the existence of 4∶0 and 3∶1/4∶0 tracts are most consistent with a G1-associated DSB. The 3∶1 tracts could reflect a G2-initiated DSB, replication of a G1-initiated nick to generate a DSB on one chromatid following DNA replication [52], or repair of a DNA molecule with a single-stranded gap [53],[54].

It is clear that DSBs, induced by X-rays or by site-specific endonucleases, stimulate both mitotic gene conversion and crossovers [55]. One argument that spontaneous mitotic recombination events are initiated by DSBs is that certain mutants that are incapable of DSB repair (such as *rad52*) are hypo-Rec [56]. Arguments in favor of other types of DNA lesions such as single-stranded nicks as recombinogenic include: 1) agents (such as UV) that result in DNA nicks, but not DSBs, are recombinogenic [55]; 2) a nick-inducing enzyme stimulates mitotic gene conversion [57]; 3) yeast strains with mutations that eliminate DSB repair grow normally [53]; 4) certain *rad52* mutants have a strong DSB repair defect, but normal rates of heteroallelic mitotic recombination [58]. The first two lines of evidence in favor of nick-initiated recombination events are not definitive since the duplication of a nicked chromosome would result in a DSB. Galli and Schiestl [52] showed that cells treated with ionizing radiation in G1 could complete mitotic recombination between direct repeats in G1, whereas G1 cells treated with ultraviolet radiation required transition through the S-period in order to complete the recombination event.

One possibility is that different types of spontaneous DNA lesions initiate different types of mitotic recombination. For example, our studies argue that spontaneous crossovers are likely to involve a DSB. In contrast, Lettier *et al.* [58] find that heteroallelic gene conversion and direct repeat recombination occur at a wild-type frequency in strains that are incapable of DSB repair. This discrepancy could be resolved by testing the effect of the *rad52* alleles used by Lettier *et al.* in our system. In addition, from an analysis of the effects of *rad51*, *rad55*, and *rad57* mutants on sister and interhomologue recombination, Mozlin *et al.* [54] argue that most sister-strand recombination reflects the repair of single-strand gaps rather than the repair of DSBs.

#### Mechanisms of generating long continuous mitotic conversion tracts

**Figure 9:**
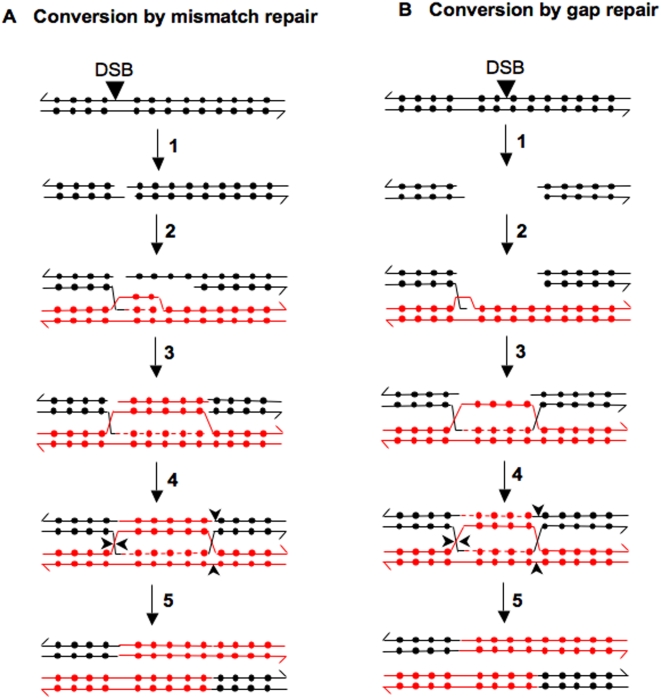
Generation of long conversion tracts by repair of mismatches within a heteroduplex or by gap repair. A) Conversion by mismatch repair. Conversion is initiated by a DSB, followed by 5′ to 3′ resection of the broken ends (step 1). The 3′ strand on one of the broken ends invades the other homologue and the invading strand is used as a primer for DNA synthesis (step 2); the newly-synthesized strand is shown as a dashed line. The broken end that is not used in the initial interaction undergoes more extensive resection. The single strand displaced by DNA synthesis pairs with the extensively-resected end, resulting in a long heteroduplex (step 3). The mismatches within the heteroduplex are converted in the same direction (excision of the black strand) to generate a long continuous conversion tract (step 4). The intermediate with double Holliday junctions is cleaved (cleavage sites indicated by arrows) to generate a conversion event associated with a crossover (step 5). B) Conversion by gap repair. Both strands of the broken ends resulting from the DSB are degraded to yield a gapped molecule (step 1). One of the ends invades the homologous chromosome and initiates DNA synthesis (step 2). The strand displaced by DNA synthesis pairs with the other broken end (step 3), and there is a second round of DNA synthesis (step 4). The intermediate is processed by cleaving the Holliday junctions as in Figure 9A (step 5).

In our study, as in previous studies, most of the conversion tracts are continuous (sites involved in conversion are not separated by sites not involved in conversion). In one version of the DSB repair model (Figure 9A), one broken end invades the other homologue, priming DNA synthesis that displaces one strand of the invaded duplex. The displaced strand forms a heteroduplex with the other resected end. Mismatches within the heteroduplex are corrected to generate the gene conversion event. One strong argument that most meiotic gene conversions reflect heteroduplex formation followed by mismatch repair is that mutants that inactivate mismatch repair reduce the frequency of gene conversion almost ten-fold and elevate the frequency of post-meiotic segregation [2]. Similar studies of the effects of mismatch repair mutants on mitotic gene conversion also demonstrate that most mitotic events are a consequence of heteroduplex formation and mismatch repair [59].

If a heteroduplex involves multiple mismatches, these mismatches must be corrected in the same direction (excision of the mismatches from the same strand) in order to observe a continuous conversion tract. Since the lengths of excision tracts in yeast are about the same as the lengths of meiotic conversion tracts (1 to 2 kb) [60], continuous conversion tracts are expected for most events. Some meiotic conversion tracts, however, are greater than 5 kb in length [18]. To explain the existence of long continuous conversion tracts, we suggested that either excision repair is targeted to one strand by some undefined mechanism or the long conversion tracts reflect a different mechanism (for example, repair of a double-stranded DNA gap) than the short tracts [60]. The same issue is raised by the very long continuous mitotic conversion tracts. One possible explanation is that mitotic gene conversion involves very long excision tracts. This possibility is unlikely based on studies of plasmids with mismatches transformed into yeast cells, demonstrating that most mitotic excision tracts are less than 1 kb [61].

A second explanation for long continuous gene conversion tracts is that they reflect repair of a double-stranded DNA gap (Figure 9B). Such gaps could arise from a processed DSB or two DSBs on the same chromosome. Although processing of DSBs occurs primarily by 5′ to 3′ degradation of one of the two strands [3], we suggest that loss of both strands, forming a gap, may occur under certain conditions (for example, a G1-induced DSB in a diploid). Orr-Weaver and Szostak [62] showed that gapped DNA molecules could be efficiently repaired, resulting in a gene conversion event. Inbar and Kupiec [63] showed that gene conversion of an HO-induced DSB was efficient even if the DSB occurred in a large heterologous insertion. One explanation of this result is that the broken ends are processed into a gap, although mechanisms that do not involve a gapped intermediate are also possible (for example, as shown in Figure 4 of Inbar and Kupiec). Finally, Zierhut and Diffley [64] have recently shown that broken DNA ends that persist into the S-period undergo degradation of both 5′ and 3′ ends, resulting in a gap.

Another mechanism in which gene conversion does not involve extensive heteroduplex formation is BIR [3],[4]. Since these events extend from the initiating DSB to the end of the chromosome, single BIR events will not lead to reciprocal exchange of a centromere-distal marker and will not produce a Can^R^ red/white sectored colony (Figure 1B). A model in which two separate BIR events can produce a long conversion tract associated with a crossover is shown in Figure S4. This process is different than the template switching described previously [65] in which a single end undergoes more than one cycle of strand invasion. Although the model shown in Figure S4 results in a long 3∶1 continuous conversion tract, this model does not explain the 4∶0 or 4∶0/3∶1 hybrid events.

One interpretation of our observations is that there are two types of mitotic gene conversion tracts, long tracts that reflect repair of a double-stranded DNA gap and shorter tracts that involve the repair of mismatches in heteroduplex DNA. Our analysis of crossover-associated conversions might be biased toward the first class, whereas studies of heteroallelic recombination might be biased to the second class of conversion. Although more than 90% of the conversion tracts were less than 30 kb in length, six exceeded this size. It is possible that these very long conversion tracts reflect a third mechanism of mitotic conversion.

### Summary

Our analysis of spontaneous mitotic crossing-over in a 120 kb *CEN5-CAN1* interval of yeast chromosome V demonstrates that most crossovers are associated with long continuous gene conversion tracts. Crossovers and conversions occur throughout the whole interval, although these events are reduced in frequency near the centromere and there is one modest hotspot for conversion located near *CAN1*. About 40% of the recombination events have properties indicative of a DSB on one homologue in G1, replication of the broken chromosome, and subsequent repair of the two broken chromatids.

## Materials and Methods

### Construction of Yeast Strains

Most of our analysis was done with two very closely related diploid strains PSL100 and PSL101; the only difference between these strains is that PSL100 is homozygous for the *ura3-1* mutation and PSL101 is heterozygous *ura3-1/URA3*. Isogenic diploids that were hemizygous for the mating type locus (PG311) or lacked *SPO11* (MD457) were also analyzed. These diploids are identical except for changes introduced by transformation. Their constructions are described in Supp. Information and Table S1. All diploids were homozygous for *ade2-1*, heterozygous for *can1-100*, and heterozygous for an insertion of *SUP4-o* at a position on chromosome V allelic to *can1-100*. As explained in Results, reciprocal crossovers between *CEN5* and *CAN1* can be selected in strains of this genotype. In addition, each diploid was derived by crossing two sequence-diverged haploids (isogenic derivatives of W303A and YJM789), resulting in a diploid with many single-nucleotide polymorphisms [15]. The homologue with the *can1-100* gene had the markers contributed by W303A and the one with the *SUP4-o* marker had the markers contributed by YJM789. As described below, we used these markers to construct a high-resolution genetic map of the *CEN5-CAN1* region.

### Genetic Analysis and Media

Standard yeast procedures were used for mating, sporulation, and tetrad dissection [14]. Rich growth medium (yeast extract, peptone, dextrose; YPD) and omission media were also made following standard recipes [14] except the medium contained 10 micrograms/ml of adenine. The solid medium used to select mitotic crossovers lacked arginine (SD-arg) and contained 120 micrograms/ml canavanine.

The diploid strains PSL100, PSL101, MD457, and PG311 were used to analyze mitotic crossovers. These strains were streaked for single colonies on YPD and incubated at 30°C. for 2 days. Individual colonies (about 20/experiment) were resuspended in 400 microliters of water. Each sample was diluted (usually by a factor of 10^5^) and plated onto solid medium lacking arginine in order to measure the number of cells per colony; colonies on the control plates were counted after the plates were incubated two days at 30°. 100 microliters of the undiluted samples were plated onto SD-arg medium containing canavanine. These plates were incubated at room temperature for four days, followed by one day of storage at 4° to minimize the background growth of canavanine-sensitive cells and accentuate the red color of colonies that lack the *SUP4-o* gene. We then counted the number of red/white sectored colonies, only counting colonies in which the smallest sector was at least one-eighth of the size of the total colony. Each sector was purified on solid YPD medium for the subsequent analysis described below.

### Physical Analysis of Markers in Sectored Colonies

We isolated yeast DNA from purified red (*can1-100/can1-100*) and white (*SUP4-o/SUP4-o*) sectors by standard procedures [14]. The numbers of sectored colonies analyzed for PSL100/101, MD457, and PG311 were 74, 14, and 14, respectively.

As described above, the diploids used in our study were heterozygous for many markers. By comparing the W303A sequence (http://www.sanger.ac.uk/gbrowse/gbrowse/cere_dmc/) and the YJM789 sequence [15], we identified 34 polymorphisms that changed restriction enzyme recognition sites that were located between *CEN5* (SGD coordinate of 152,000) and *can-100/SUP4-o* (SGD coordinate of about 32,000). The positions of these polymorphisms (SGD coordinates) are shown in Table S2. For each polymorphism analyzed for individual sectors, we PCR-amplified the genomic DNA using the primers that flanked the polymorphism (Table S2) and treated the resulting DNA fragment with the relevant restriction enzyme. The products were analyzed by standard agarose gel electrophoresis. This analysis allowed us to determine whether the strain representing the red or white portion of the sectored colony was homozygous for the YJM789 polymorphism, homozygous for the W303A polymorphism, or heterozygous for the polymorphism. Additional details of our analysis are given in Supp. Information.

### Physical Analysis of Markers in Meiotic Products

The meiotic segregation of markers in the diploid PSL101 was examined in 21 tetrads. All four spores of each tetrad were examined. All 34 markers were analyzed in six of the tetrads; the analysis of the remaining 15 was done by the same approach used for most of the mitotic sectors. In each tetrad, the crossovers and gene conversion events were mapped to the highest degree of resolution possible with the 34 markers.

### Statistical Analysis

Statistical analyses (Fisher exact test, Chi-square tests, and linear correlation analysis) were done using the VassarStats Website (http://faculty.vassar.edu/lowry/VassarStats.html).

## Supporting Information

Figure S1 Patterns of conversion and crossing over that generate one of the exceptional classes of sectored colonies. In this diagram, the W303A markers are shown as red circles and the YJM789 markers are shown as black circles; the centromere is shown as a white circle or oval. The direction of conversion is indicated by the small arrow. As explained in the text and as shown in Figure 4B, if the W303A-derived chromosome is the donor in a conversion event, at the site of conversion, we expect that the red sector will be homozygous for the W303A-derived marker and the white sector will be heterozygous. About 5% of the sectored colonies had the reverse arrangement (shown at the bottom of this figure). This configuration can be explained by the following sequence of events. One chromosome is broken in G1, and replicated to yield two broken chromatids. The DSB on chromatid 2 is repaired by an interaction with chromatid 3, resulting in a crossover, but no conversion (Step 1). The DSB on chromatid 1 is repaired using sequences derived from chromatid 3 (as shown) or 4; this repair event is associated with a conversion of one marker, but no crossover (Step 2). Chromatids 1 and 3 segregate to one daughter cell, and chromatids 2 and 4 segregate to the other, generating the red/white sectored colony (Step 3).
(0.17 MB TIF)

Figure S2 Patterns of conversion and crossing over required to generate a conversion tract with a crossover in the middle of the tract. As in Figure S1, a broken chromosome is replicated to yield two broken chromatids. Chromatid 2 is repaired by an interaction with chromatid 3 associated with a conversion of a centromere-distal marker and a crossover (Step 1). Chromatid 1 is repaired by an interaction with chromatid 3 (as shown) or 4. This repair event is associated with a conversion of a centromere-proximal marker, but no crossover (Step 2). Chromatids 1 and 3 co-segregate, as do chromatids 2 and 4 (Step 3).
(0.18 MB TIF)

Figure S3 Model proposed by Esposito [7] to explain G1-initiated mitotic recombination. Dotted lines in this figure represent single strands of a DNA duplex. Derived from a strain with *trp5* heteroalleles and a distal heterozygous marker, Esposito [7] observed Trp^+^ colonies that had homozygous sectors for the distal marker. To explain such sectors, he suggested that an asymmetric heteroduplex is formed in G1 that includes both of the heteroallelic markers. Repair of both resulting mismatches using wild-type information would result in a wild type allele. The resulting intermediate with an unresolved Holliday junction would be replicated to produce the RCO. Resolution of the Holliday junction in G1 would not produce a reciprocal crossover.
(0.07 MB TIF)

Figure S4 Mitotic conversion tracts with associated crossover generated by a double BIR event. The broken DNA in the black chromatid invades and begins to replicate the red chromatid (step 1). After region B of the chromosome has been replicated, the replication fork breaks (step 2), and the broken end invades the black chromatid (step 3). Completion of DNA synthesis results in a long conversion tract with a flanking RCO (step 4). The acentric chromatid fragment with the B and C regions is lost.
(0.07 MB TIF)

Table S1 Primers used in strain constructions.
(0.05 MB DOC)

Table S2 Primers used in analysis of polymorphic markers. ^1^As described in the text, we identified sequence differences between two yeast strains (W303a and YJM789) that altered restriction sites in the region between *CEN5* and *CAN1*. We examined the segregation of these sites by generating short PCR fragments that included the sites, and treating the resulting fragments with restriction enzymes that cut the DNA derived from one strain, but not the other. ^2^The position of the polymorphism is indicated in coordinates based on the Stanford Genome Database. The numbers in parentheses represent the abbreviations of the coordinates used in the figures. ^3^This column indicates the enzymes used to diagnose the polymorphism. The enzyme written in boldface has a recognition site at the diagnostic position in YJM789, but not in W303a. The enzyme written in plain face has a recognition site in W303a, but not in YJM789.
(0.16 MB DOC)

Table S3 Lengths of mitotic conversion tracts in PSL100/PSL101. ^1^The maximum, minimum, and average lengths of mitotic gene conversion tracts were calculated as described in the text. The table is ordered by the average length of the conversion events, beginning with the shortest. ^2^In this column, we indicate whether the conversion tract was a 3∶1 tract (1), a 4∶0 tract (2), or a hybrid 3∶1, 4∶0 tract (3).
(0.10 MB DOC)

Table S4 Lengths of mitotic conversion tracts in MD457. ^1^The maximum, minimum, and average lengths of mitotic gene conversion tracts were calculated as described in the text. The table is ordered by the average length of the conversion events, beginning with the shortest. ^2^In this column, we indicate whether the conversion tract was a 3∶1 tract (1), a 4∶0 tract (2), or a hybrid 3∶1, 4∶0 tract (3).
(0.05 MB DOC)

Table S5 Lengths of mitotic conversion tracts in PG311. ^1^The maximum, minimum, and average lengths of mitotic gene conversion tracts were calculated as described in the text. The table is ordered by the average length of the conversion events, beginning with the shortest. ^2^In this column, we indicate whether the conversion tract was a 3∶1 tract (1), a 4∶0 tract (2), or a hybrid 3∶1, 4∶0 tract (3).
(0.05 MB DOC)

Table S6 Lengths of meiotic conversion tracts in PSL101. ^1^The maximum, minimum, and average lengths of meiotic gene conversion tracts were calculated as described in the text. The table is ordered by the average length of the conversion events, beginning with the shortest.
(0.04 MB DOC)

Text S1 A fine-structure map of spontaneous mitotic crossovers in the yeast *Saccharomyces cerevisiae*.
(0.05 MB DOC)
