# Supplementary figures and images for: A Fine-Structure Map of Spontaneous Mitotic Crossovers in the Yeast Saccharomyces cerevisiae
Source: PLoS Genet. 2009 Mar 13;5(3):e1000410. doi: 10.1371/journal.pgen.1000410 (PMC2646836; doi:10.1371/journal.pgen.1000410)

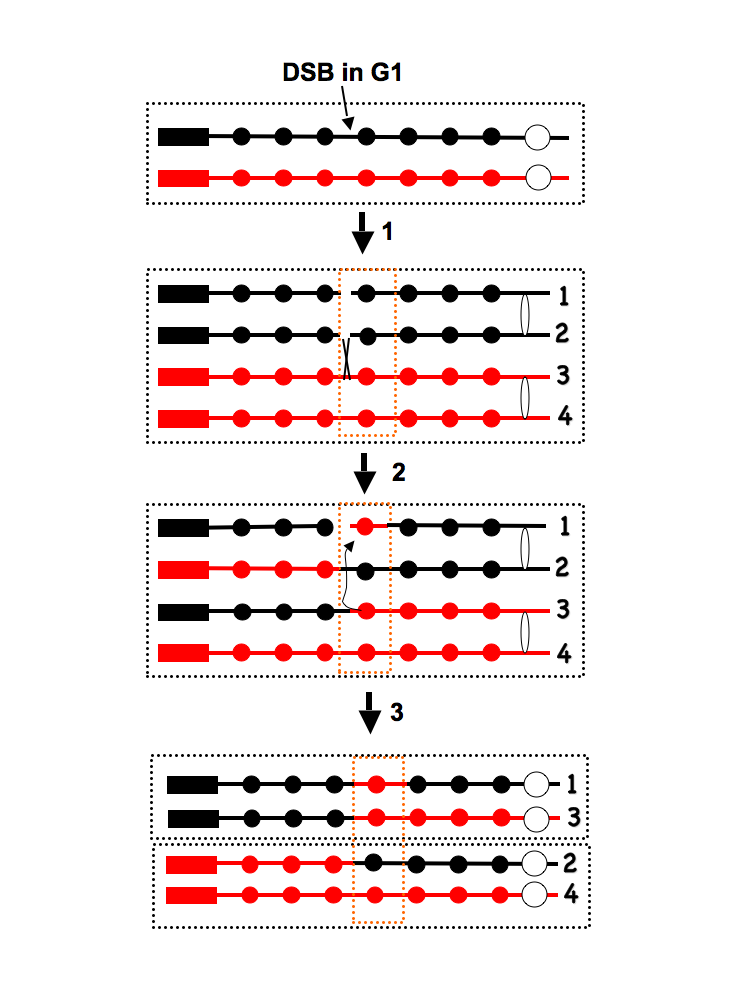

Supplement: Figure S1 — Patterns of conversion and crossing over that generate one of the exceptional classes of sectored colonies. In this diagram, the W303A markers are shown as red circles and the YJM789 markers are shown as black circles; the centromere is shown as a white circle or oval. The direction of conversion is indicated by the small arrow. As explained in the text and as shown in Figure 4B, if the W303A-derived chromosome is the donor in a conversion event, at the site of conversion, we expect that the red sector will be homozygous for the W303A-derived marker and the white sector will be heterozygous. About 5% of the sectored colonies had the reverse arrangement (shown at the bottom of this figure). This configuration can be explained by the following sequence of events. One chromosome is broken in G1, and replicated to yield two broken chromatids. The DSB on chromatid 2 is repaired by an interaction with chromatid 3, resulting in a crossover, but no conversion (Step 1). The DSB on chromatid 1 is repaired using sequences derived from chromatid 3 (as shown) or 4; this repair event is associated with a conversion of one marker, but no crossover (Step 2). Chromatids 1 and 3 segregate to one daughter cell, and chromatids 2 and 4 segregate to the other, generating the red/white sectored colony (Step 3). (0.17 MB TIF) [file pgen.1000410.s001.tif]

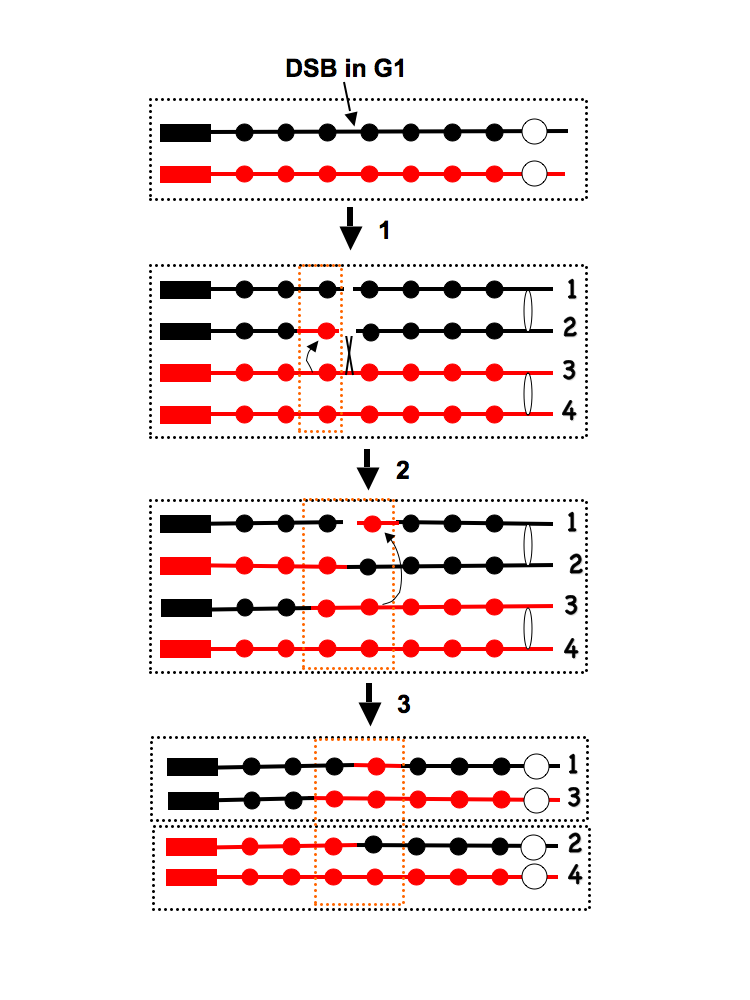

Supplement: Figure S2 — Patterns of conversion and crossing over required to generate a conversion tract with a crossover in the middle of the tract. As in Figure S1, a broken chromosome is replicated to yield two broken chromatids. Chromatid 2 is repaired by an interaction with chromatid 3 associated with a conversion of a centromere-distal marker and a crossover (Step 1). Chromatid 1 is repaired by an interaction with chromatid 3 (as shown) or 4. This repair event is associated with a conversion of a centromere-proximal marker, but no crossover (Step 2). Chromatids 1 and 3 co-segregate, as do chromatids 2 and 4 (Step 3). (0.18 MB TIF) [file pgen.1000410.s002.tif]

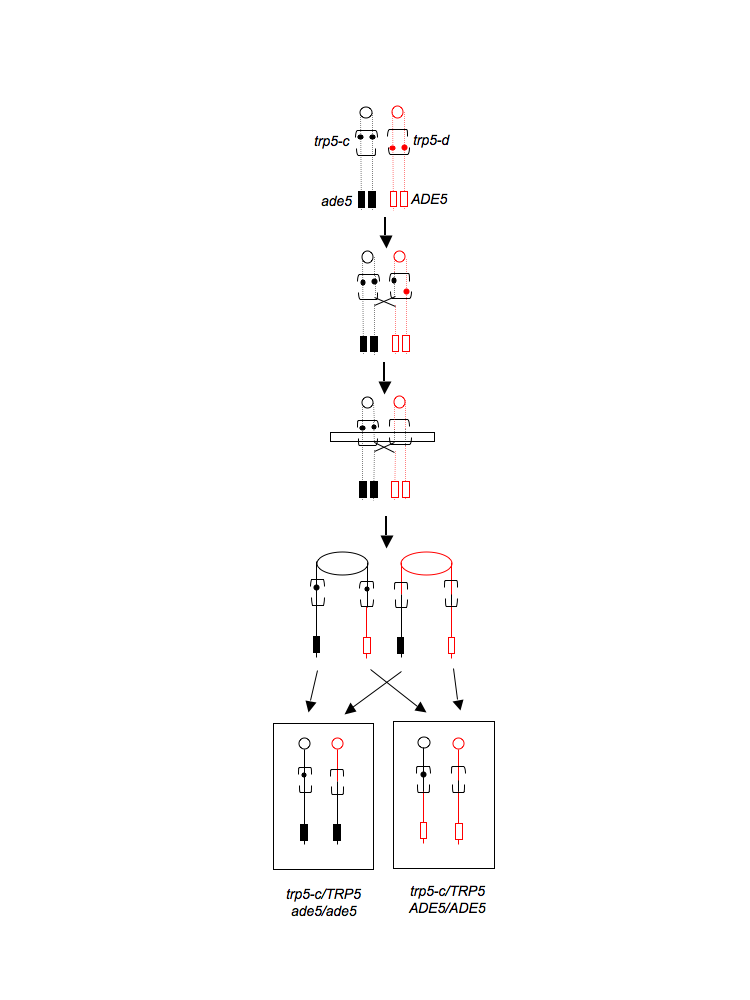

Supplement: Figure S3 — Model proposed by Esposito [7] to explain G1-initiated mitotic recombination. Dotted lines in this figure represent single strands of a DNA duplex. Derived from a strain with trp5 heteroalleles and a distal heterozygous marker, Esposito [7] observed Trp+ colonies that had homozygous sectors for the distal marker. To explain such sectors, he suggested that an asymmetric heteroduplex is formed in G1 that includes both of the heteroallelic markers. Repair of both resulting mismatches using wild-type information would result in a wild type allele. The resulting intermediate with an unresolved Holliday junction would be replicated to produce the RCO. Resolution of the Holliday junction in G1 would not produce a reciprocal crossover. (0.07 MB TIF) [file pgen.1000410.s003.tif]

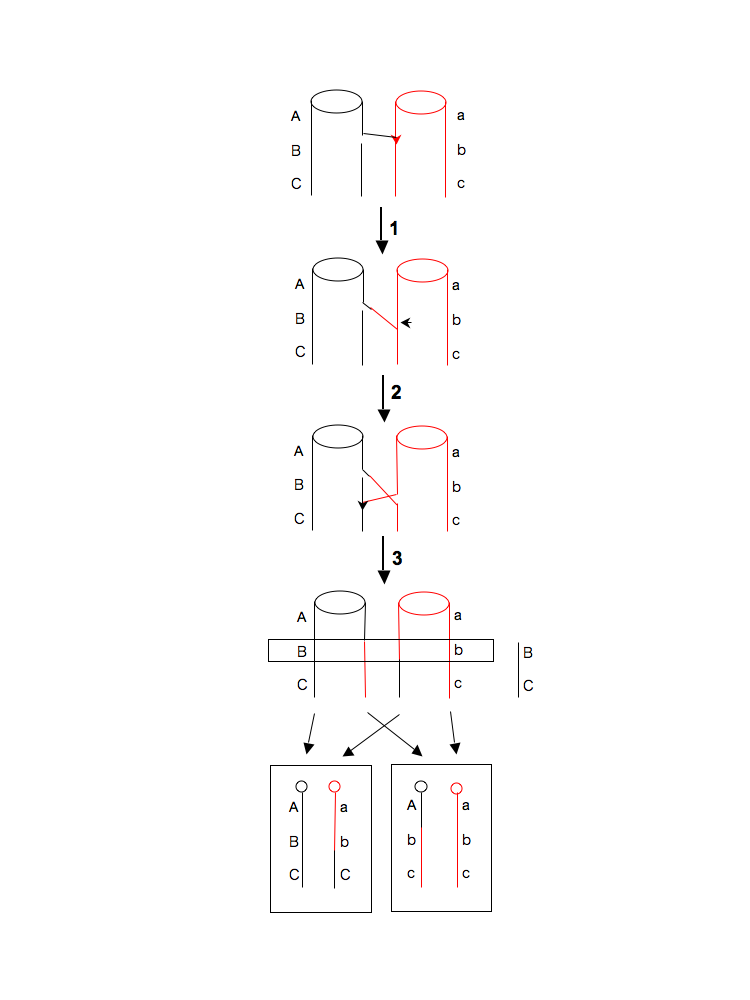

Supplement: Figure S4 — Mitotic conversion tracts with associated crossover generated by a double BIR event. The broken DNA in the black chromatid invades and begins to replicate the red chromatid (step 1). After region B of the chromosome has been replicated, the replication fork breaks (step 2), and the broken end invades the black chromatid (step 3). Completion of DNA synthesis results in a long conversion tract with a flanking RCO (step 4). The acentric chromatid fragment with the B and C regions is lost. (0.07 MB TIF) [file pgen.1000410.s004.tif]
