# Supplementary material for: A Fine-Structure Map of Spontaneous Mitotic Crossovers in the Yeast Saccharomyces cerevisiae
Source: PLoS Genet. 2009 Mar 13;5(3):e1000410. doi: 10.1371/journal.pgen.1000410 (PMC2646836; doi:10.1371/journal.pgen.1000410)
Supplement: Table S1 — Primers used in strain constructions. (0.05 MB DOC) [file pgen.1000410.s005.doc]

| **Table S1. Primers used in strain constructions.** | |  |  |  |  |  |
| --- | --- | --- | --- | --- | --- | --- |
|  |  |  |  |  |  |  |
| **Primer name** | **Sequence (5’ to 3’)** |  |  |  |  |  |
| ADE2-KIURA-381 | ACTCCAATGACCACGTTAATGGCTCCTTTTCCAATCCTTTGATATCGAAAAACTAGCTGTGGTTATTCGTGGATCTATATC | | | | | |
| [ADE2-KIURA3 DS](http://www.idtdna.com/OrderStatus/SpecSheet.aspx?OrderNum=1783789&MfgID=25075043&MfgLocID=1&SearchDays=&SearchNum=1783789&SearchPO=&SearchRef=&ProdID=1) | GATTCTTTAGTGTAGGAACATCAACATGCTCAATCTCAATCGTTAGCACATCACATTTTTTTAAACTCTTTTTCGATGAT | | | | | |
| [ADE2-11](http://www.idtdna.com/OrderStatus/SpecSheet.aspx?OrderNum=1783789&MfgID=25076521&MfgLocID=1&SearchDays=&SearchNum=1783789&SearchPO=&SearchRef=&ProdID=1213) | GAACAGTTGGTATATTAGGAGGGGGAC |  |  |  |  |  |
| [ADE2-392](http://www.idtdna.com/OrderStatus/SpecSheet.aspx?OrderNum=1783789&MfgID=25075413&MfgLocID=1&SearchDays=&SearchNum=1783789&SearchPO=&SearchRef=&ProdID=1213) | TCACTGGCTTGTTCCACAGGAACAC |  |  |  |  |  |
| ADE2 DS | CAAGACGAATGGAAAACCCAAATCTC |  |  |  |  |  |
| [CAN1-801](http://www.idtdna.com/OrderStatus/SpecSheet.aspx?OrderNum=1829310&MfgID=25674695&MfgLocID=1&SearchDays=&SearchNum=1829310&SearchPO=&SearchRef=&ProdID=1213) | CGAATCAGGGAATCCCTTTTTGC |  |  |  |  |  |
| [CAN1-2974](http://www.idtdna.com/OrderStatus/SpecSheet.aspx?OrderNum=1829310&MfgID=25674045&MfgLocID=1&SearchDays=&SearchNum=1829310&SearchPO=&SearchRef=&ProdID=1213) | CTGAAGGAGTTTCAAATGCTTC |  |  |  |  |  |
| CAN1-0901 | GTTGGATCCAGTTTTTAATCTGTC |  |  |  |  |  |
| CAN1-2800 | GTGATCAAAGGTAATAAAACGTC |  |  |  |  |  |
| [URA3 US](https://www.idtdna.com/OrderStatus/SpecSheet.aspx?OrderNum=4205333&MfgID=29331200&MfgLocID=1&SearchDays=&SearchNum=4205333&SearchPO=&SearchRef=&ProdID=1213) | ACGCATATGTGGTGTTGAAGAAAC |  |  |  |  |  |
| [URA3 DS](https://www.idtdna.com/OrderStatus/SpecSheet.aspx?OrderNum=4205333&MfgID=29331401&MfgLocID=1&SearchDays=&SearchNum=4205333&SearchPO=&SearchRef=&ProdID=1213) | GTTGTTCTTTGGAGTTCAATGCGTC |  |  |  |  |  |
| MATALPHA NATF | ATATATATATATATATTCTACACAGATATATACATATTTGTTTTTCGGGCCGTACGCTGAAGGTCGAC | | | | | |
| MATALPHA NATR | TGAACAACATTCAGTACTCGAAAGATAAACAACCTCCGCCACGACCACACTCATCGATGAATTCGAGCTCG | | | | | |
| SPO11NATF | ATAACCTCACATATTTGTCTTCACCCTTAAGATTTTACGATTTACTAAGTTCACCTTCTCCGTACGCTGCAGGTCGAC | | | | | |
| SPO11NATR | CGTTTTCAATTCTTGAAAAACATTTTTTATAAAGCAACAGCTCCCATTCTTATTCATTTATCGATGAATTCGAGCTCG | | | | | |
| nat-R | GTCGACCTGCAGCGTACG | | | | | |
| V-63628 | GATTGCAGTTGCCCGAAAACTGT |  |  |  |  |  |
