# Supplementary material for: A Fine-Structure Map of Spontaneous Mitotic Crossovers in the Yeast Saccharomyces cerevisiae
Source: PLoS Genet. 2009 Mar 13;5(3):e1000410. doi: 10.1371/journal.pgen.1000410 (PMC2646836; doi:10.1371/journal.pgen.1000410)
Supplement: Table S2 — Primers used in analysis of polymorphic markers. 1As described in the text, we identified sequence differences between two yeast strains (W303a and YJM789) that altered restriction sites in the region between CEN5 and CAN1. We examined the segregation of these sites by generating short PCR fragments that included the sites, and treating the resulting fragments with restriction enzymes that cut the DNA derived from one strain, but not the other. 2The position of the polymorphism is indicated in coordinates based on the Stanford Genome Database. The numbers in parentheses represent the abbreviations of the coordinates used in the figures. 3This column indicates the enzymes used to diagnose the polymorphism. The enzyme written in boldface has a recognition site at the diagnostic position in YJM789, but not in W303a. The enzyme written in plain face has a recognition site in W303a, but not in YJM789. (0.16 MB DOC) [file pgen.1000410.s006.doc]

| **Table S2. Primers used in analysis of polymorphic markers**^1^ | | | |
| --- | --- | --- | --- |
| **SGD coordinates for polymorphic site**^2^ | **Primer name** | **Primer sequence (5’ to 3’)** | **Diagnostic restriction enzyme**^3^ |
|  |  |  |  |
| **34,812 (35)** | V-34290 | GTTGCCTGTATCTCCGAATAGGAC | *Taq*I |
|  | V-35030 | CAGGTCTTGGAAAATTCTCATCAAT |  |
|  |  |  |  |
| **40,688 (41)** | V-40455 | TTTAAACCTGGGGCGAAAACGTC | ***Hpa*II** |
|  | V-40876 | ACTGTGCCGGTACTATCGCTT |  |
|  |  |  |  |
| **43,075 (43)** | V-42860 | TGTCTGCTTCCTTACTTGGCTG | ***Alu*I** |
|  | V-43242 | GCTTACTGCTGACCTTTGCCTT |  |
|  |  |  |  |
| **44,403 (44)** | V-44164 | GTGTTCTACTATGTCGACTC | *Alu*I |
|  | V-44532 | AGCTAACGTATCACCATTTAAC |  |
|  |  |  |  |
| **46,347 (46)** | V-46267 | CGATTGACGTAAACGGTAATAAATGT | *Hha*I |
|  | V-46590 | ATTTATAAGACAAAAAGTTAGAATTCT |  |
|  |  |  |  |
| **48,898 (49)** | [V-48700](https://www.idtdna.com/OrderStatus/SpecSheet.aspx?OrderNum=4903343&MfgID=39489100&MfgLocID=1&SearchDays=&SearchNum=4903343&SearchPO=&SearchRef=&ProdID=1213) | TTAGAAGTCAGCATCAGCTTGTG | *Mse*I |
|  | V-49179 | GCTTTCTGACGACGGTGGAGA |  |
|  |  |  |  |
| **51,707 (52)** | V-51560 | TGCACTTGTGGAAAGAATCGCC | *Dra*I |
|  | V-51985 | CCTTCAAGGCTACTTTCAGATGC |  |
|  |  |  |  |
| **54,915 (55)** | V-54657 | TTTCAAAAAGGTTTCTAAGTGGTGAC | *Rsa*I |
|  | V-55143 | CTACCAGTTTGCCAAATTCTTCAAC |  |
|  |  |  |  |
| **56,166 (56)** | V-55825 | CTATCCGCTATGGCTGCTAC | *Hha*I |
|  | V-56394 | AAGACAAAGGAACTCGGCAC |  |
|  |  |  |  |
| **57,448 (57)** | V-57125 | CGATACGGTTCCATTTTTGGCCGCA | ***Hpa*II** |
|  | V-57682 | GGCTCACCACACAACTGCTTGA |  |
|  |  |  |  |
| **60,163 (60)** | V-59896 | CCAAGAGATCTGTTTTGATGGTC | *Hpy*188III |
|  | V-60417 | TGGTGGCCATAGAAATAGAACG |  |
|  |  |  |  |
| **63,936 (64)** | V-63638 | ATTGAACTGCTTTGGTTACCTCTC | *Hae*III |
|  | V-64114 | CCTTCATTAAATTGGCGTTTGTCTC |  |
|  |  |  |  |
| **70,336 (70)** | V-70090 | GCCAAGAAGTACTGCGATGTTAC | ***Hha*I** |
|  | V-70583 | TCTTCAAAGTGGAGACGATGCTG |  |
|  |  |  |  |
| **76,383 (76)** | V-76193 | GTTGATACTGCATACGATGTAAGGC | *Dra*I |
|  | V-76567 | CGGCGGAACATCTTTCGTGAATATA |  |
|  |  |  |  |
| **80,094 (80)** | [V-79811](https://www.idtdna.com/OrderStatus/SpecSheet.aspx?OrderNum=4205333&MfgID=29331399&MfgLocID=1&SearchDays=&SearchNum=4205333&SearchPO=&SearchRef=&ProdID=1213) | TAACCCTTTACCTGACCTGAATGTC | ***BgI*II** |
|  | [V-80476](https://www.idtdna.com/OrderStatus/SpecSheet.aspx?OrderNum=4205333&MfgID=29331400&MfgLocID=1&SearchDays=&SearchNum=4205333&SearchPO=&SearchRef=&ProdID=1213) | GTAATTGTCTCCCATTTTTGGTATAC |  |
|  |  |  |  |
| **82,767 (83)** | V-82592 | TAAGCTAACCATTTTTCTATTG | Rsa*I* |
|  | V-82962 | GGTCTTTAACTTTTCTTTGAGTG |  |
|  |  |  |  |
| **86,772 (87)** | V-86400 | [GTGTAATTCATTGGGGAGGATGA](https://www.idtdna.com/OrderStatus/SpecSheet.aspx?OrderNum=4440307&MfgID=32910362&MfgLocID=1&SearchDays=&SearchNum=&SearchPO=&SearchRef=&ProdID=1213) | ***Alu*I** |
|  | V-86943 | CTTCCATAATTGACGTTTGTATC |  |
|  |  |  |  |
| **91,715 (92)** | V-91473 | TTGATTTTCGCTGTTATTGCATCC | *Taq*I |
|  | V-91953 | TACTGTTTTTTCTTTCGACAGCCC |  |
|  |  |  |  |
| **94,329 (94)** | [V-94058](http://www.idtdna.com/OrderStatus/SpecSheet.aspx?OrderNum=1983273&MfgID=28951905&MfgLocID=1&SearchDays=&SearchNum=1983273&SearchPO=&SearchRef=&ProdID=1213) | GTTTATATTTTGTTGCTAGCGTTACGG | ***Taq*I** |
|  | [V-94699](http://www.idtdna.com/OrderStatus/SpecSheet.aspx?OrderNum=1983273&MfgID=28952314&MfgLocID=1&SearchDays=&SearchNum=1983273&SearchPO=&SearchRef=&ProdID=1213) | CGGGTCAGAATACGAAGTATATTATG |  |
|  |  |  |  |
| **99,267 (99)** | V-98931 | ATACTGTTATCGAAACTACGGGC | *Eco*RV |
|  | V-99436 | TGTGTGCATGGTTATGTAGATTG |  |
|  |  |  |  |
| **103,991 (104)** | [V-103659](https://www.idtdna.com/OrderStatus/SpecSheet.aspx?OrderNum=4439022&MfgID=32893850&MfgLocID=1&SearchDays=&SearchNum=4439022&SearchPO=&SearchRef=&ProdID=1213) | GAGTTTTGTTCTGGCCACAGTGGC | ***Hha*I** |
|  | [V- 104202](https://www.idtdna.com/OrderStatus/SpecSheet.aspx?OrderNum=4439022&MfgID=32893851&MfgLocID=1&SearchDays=&SearchNum=4439022&SearchPO=&SearchRef=&ProdID=1213) | GAGAGTGGATTTAGAGATTCATTCGT |  |
|  |  |  |  |
| **107,884 (108)** | [V-107668](https://www.idtdna.com/OrderStatus/SpecSheet.aspx?OrderNum=4205333&MfgID=29331198&MfgLocID=1&SearchDays=&SearchNum=4205333&SearchPO=&SearchRef=&ProdID=1213) | TTGAAGGACCCACAGACCGATGC | ***Alu*I** |
|  | [V-108097](https://www.idtdna.com/OrderStatus/SpecSheet.aspx?OrderNum=4205333&MfgID=29331199&MfgLocID=1&SearchDays=&SearchNum=4205333&SearchPO=&SearchRef=&ProdID=1213) | GTGATTTTCGTTCCTTTTTGAGCG |  |
|  |  |  |  |
| **111,516 (112)** | V-111339 | [CACTGCTTGTAAACCATAGAC](https://www.idtdna.com/OrderStatus/SpecSheet.aspx?OrderNum=4627976&MfgID=35819052&MfgLocID=1&SearchDays=&SearchNum=&SearchPO=&SearchRef=&ProdID=1213) | ***Rsa*I** |
|  | V-111719 | GGCTGCAATATGGTTAGTGAATC |  |
|  |  |  |  |
| **113,600 (114)** | V-113331 | GGGTCGATGAAGCTATTAGAA | ***Msp*I** |
|  | V-113830 | CAAAAGCTCCAAGGGTGTTA |  |
|  |  |  |  |
| **115,035 (115)** | V-114800 | [CGGAGTACTTGTCCAAATTAA](https://www.idtdna.com/OrderStatus/SpecSheet.aspx?OrderNum=4627976&MfgID=35819050&MfgLocID=1&SearchDays=&SearchNum=4627976&SearchPO=&SearchRef=&ProdID=1213) | *Taq*I |
|  | V-115159 | CTGTCAATTTCTTGTATTCTA |  |
|  |  |  |  |
| **117,289 (117)** | V-117099 | AAAGAAAAAGCTTCATGGCC | ***Hha*I** |
|  | V-117498 | TAGATATATATACGCCAGTAC |  |
|  |  |  |  |
| **118,783 (119)** | V-118624 | CGCGGTTTATTCTGCCAGGC | ***Hha*I** |
|  | V-119048 | AACGCGACTATGGGGATTGG |  |
|  |  |  |  |
| **122,334 (122)** | [V-122161](http://www.idtdna.com/OrderStatus/SpecSheet.aspx?OrderNum=1983273&MfgID=28953400&MfgLocID=1&SearchDays=&SearchNum=1983273&SearchPO=&SearchRef=&ProdID=1213) | AGCGTTCATGAACTGCAGCTGATTC | ***Hha*I** |
|  | [V-122498](http://www.idtdna.com/OrderStatus/SpecSheet.aspx?OrderNum=1983273&MfgID=28954280&MfgLocID=1&SearchDays=&SearchNum=1983273&SearchPO=&SearchRef=&ProdID=1213) | CTCTTCGTTTTGTTTGTCCCGTTC |  |
|  |  |  |  |
| **125,754 (126)** | V-125473 | ACCAATTCTGGCCTATCTTTAAGC | ***Xho*I** |
|  | V-126033 | TAGATCAAATACTTACTTCAACGGG |  |
|  |  |  |  |
| **133,080 (133)** | V-132837 | [ACCCCTTTTTGCCTATATTAC](https://www.idtdna.com/OrderStatus/SpecSheet.aspx?OrderNum=4637883&MfgID=35970226&MfgLocID=1&SearchDays=&SearchNum=&SearchPO=&SearchRef=&ProdID=1213) | ***Dde*I** |
|  | V-133228 | [ACAACCACTTGTCAGCTAGG](https://www.idtdna.com/OrderStatus/SpecSheet.aspx?OrderNum=4637883&MfgID=35970227&MfgLocID=1&SearchDays=&SearchNum=&SearchPO=&SearchRef=&ProdID=1213) |  |
|  |  |  |  |
| **140,703 (141)** | V-140520 | GCACTTTATTCCCCGAAGATCTTC | ***Hpy*CH4V** |
|  | V-140946 | CAAACGTGGGGGTATAACTACAATC |  |
|  |  |  |  |
| **144,265 (144)** | V-143365 | CCAATAATAATAGTGCAAGCTCTGC | *Hin*fI |
|  | V-144023-R | GGCAAATGAAGATGAAATTAAAGGCA |  |
|  |  |  |  |
| **146,855 (147)** | V-146424 | TACAATTAGTAGAAGCCCTTTGC | ***Hpa*II** |
|  | V-147181 | CGACAGTAATGACATAAACGTG |  |
|  |  |  |  |
| **151,440 (151)** | V-151173 | CCCGGAATACATCGTGTAGTC | ***Hin*fI** |
|  | V-151638 | ATTCAATGACAGAAACATTACGAAG |  |
