# Supplementary material for: A Fine-Structure Map of Spontaneous Mitotic Crossovers in the Yeast Saccharomyces cerevisiae
Source: PLoS Genet. 2009 Mar 13;5(3):e1000410. doi: 10.1371/journal.pgen.1000410 (PMC2646836; doi:10.1371/journal.pgen.1000410)
Supplement: Table S4 — Lengths of mitotic conversion tracts in MD457. 1The maximum, minimum, and average lengths of mitotic gene conversion tracts were calculated as described in the text. The table is ordered by the average length of the conversion events, beginning with the shortest. 2In this column, we indicate whether the conversion tract was a 3∶1 tract (1), a 4∶0 tract (2), or a hybrid 3∶1, 4∶0 tract (3). (0.05 MB DOC) [file pgen.1000410.s008.doc]

Table S4. Lengths of mitotic conversion tracts in MD457^1^

| **Sectored colony # (MD457)** | **Type of conversion tract** | **Max. Length (bp)** | **Min. Length (bp)** | **Ave. Length (bp)** |
| --- | --- | --- | --- | --- |
|  |  |  |  |  |
| 2-2 | 1 | 8210 | 3272 | 5741 |
| 4-3 | 1 | 14949 | 1 | 7475 |
| 2-9 | 3 | 13555 | 4724 | 9139.5 |
| 2-6 | 2 | 16158 | 6047 | 11102.5 |
| 2-12 | 1 | 18045 | 9587 | 13816 |
| 2-5 | 2 | 24676 | 15421 | 20048.5 |
| 4-2 | 3 | 23248 | 17088 | 20168 |
| 2-7 | 3 | 31552 | 22836 | 27194 |
| 2-13 | 1 | 33308 | 25933 | 29620.5 |
| 2-10 | 1 | 35562 | 27068 | 31315 |
| 2-8 | 1 | 41572 | 29648 | 35610 |
| 2-3 | 1 | 107237 | 98269 | 102753 |
