# Supplementary material for: A Fine-Structure Map of Spontaneous Mitotic Crossovers in the Yeast Saccharomyces cerevisiae
Source: PLoS Genet. 2009 Mar 13;5(3):e1000410. doi: 10.1371/journal.pgen.1000410 (PMC2646836; doi:10.1371/journal.pgen.1000410)
Supplement: Table S6 — Lengths of meiotic conversion tracts in PSL101. 1The maximum, minimum, and average lengths of meiotic gene conversion tracts were calculated as described in the text. The table is ordered by the average length of the conversion events, beginning with the shortest. (0.04 MB DOC) [file pgen.1000410.s010.doc]

Table S6. Lengths of meiotic conversion tracts in PSL101^1^

| **PSL101 tetrad #** | **Max. Length (bp)** | **Min. Length (bp)** | **Ave. Length (bp)** |
| --- | --- | --- | --- |
|  |  |  |  |
| 31 | 3715 | 1 | 1858 |
| 27 | 3715 | 1 | 1858 |
| 29 | 3997 | 1 | 1999 |
| 9 | 3997 | 1 | 1999 |
| 10 | 9758 | 1 | 4880 |
| 33 | 10746 | 1 | 5374 |
| 28 | 12447 | 1 | 6224 |
| 13 | 12447 | 1 | 6224 |
| 14 | 11621 | 4005 | 7813 |
| 37 | 14297 | 3420 | 8859 |
