# Supplementary material for: A Fine-Structure Map of Spontaneous Mitotic Crossovers in the Yeast Saccharomyces cerevisiae
Source: PLoS Genet. 2009 Mar 13;5(3):e1000410. doi: 10.1371/journal.pgen.1000410 (PMC2646836; doi:10.1371/journal.pgen.1000410)
Supplement: Text S1 — A fine-structure map of spontaneous mitotic crossovers in the yeast Saccharomyces cerevisiae. (0.05 MB DOC) [file pgen.1000410.s011.doc]

**Text S1**

**A Fine-structure Map of Spontaneous Mitotic Crossovers in the Yeast *Saccharomyces cerevisiae***

Phoebe S. Lee, Patricia W. Greenwell, Margaret Dominska, Malgorzata Gawel, Monica Hamilton, and Thomas D. Petes

**Supplementary Materials and Methods**

**Construction of yeast strains.**

The diploid PSL100 was constructed by crossing the haploids PSL2 and PSL5. PSL2 (*MATa ade2-1 can1-100 ura3-1 trp1-1 V9229::HYG V261553::LEU2*) was derived from sporulation of the diploid MAB6 [1] and is isogenic with W303A [2] except for markers introduced by transformation. The markers *V9229::HYG* and *V261553::LEU2* are insertions of genes encoding hygromycin resistance and *LEU2* into chromosome V at the Stanford Genome Database (SGD) coordinates 9229 and 261553, respectively.

The PSL5 haploid (*MATα ade2-1ura3 can1Δ::SUP4-o gal2 ho::hisG*) was constructed in several steps. The haploid YJM799 (*MATα ura3 gal2 ho::hisG*) is isogenic to YJM789 (except for changes introduced by transformation or crosses with isogenic strains) and was provided by John McCusker (Department of Molecular Genetics and Microbiology, Duke University School of Medicine); YJM789 was derived from a clinical yeast isolate and its genome has recently been sequenced [3]. The *ADE2* gene in YJM799 was disrupted by transformation with a DNA fragment obtained by PCR amplification of the plasmid pCORE [4] with the primers ADE2-KlURA-381 and ADE2-KlURA DS; all primer sequences used in strain constructions are in Table S1. The transforming fragment contained the selectable markers *K. lactis URA3* and *KANMX* with 60 bp of flanking homology to the *ADE2* gene. The resulting Ura^+^ red transformant was PSL3. The *URA3/KANMX* insertion in PSL3 was replaced by the *ade2-1* allele by transformation of PSL3 with a DNA fragment obtained by PCR amplification of genomic DNA of strain MD235 [1] with the primers ADE2-392 and ADE2-11. The replacement of the *URA3/KANMX* insertion with *ade2-1* (an ochre mutation in *ADE2*) was selected using 5-fluoro-orotate (5-FOA)-containing medium [5]; the resulting haploid was PSL4. The insertion of *ade2-1* was confirmed by sequencing a PCR fragment generated using PSL4 genomic DNA and the primers ADE2-11 and ADE2 DS. The last step in the construction was the replacement of the *CAN1* gene of PSL4 with *SUP4-o* (an ochre-suppressing tRNA). PSL4 was transformed with a DNA fragment containing the *SUP4-o* and flanking sequences derived from the *CAN1* locus, generated by amplification of genomic DNA of MD242-1 [1] with primers CAN1-801 and CAN1-2974. The canavanine-resistant transformant that was Ade^+^ and formed white colonies (indicating suppression of the *ade2-1* allele) was called PSL5. The construction was confirmed by sequencing a PCR fragment generated using primers CAN1-901 and CAN1-2800 and genomic DNA of PSL5.

The diploid PSL100 was made by crossing PSL2 and PSL5. The closely-related diploid PSL101 was constructed by transforming PSL100 with a fragment generated by amplifying genomic DNA of the strain YJM780 (a *URA3* strain otherwise isogenic with YJM799; provided by John McCusker) with the primers URA3 US and URA3 DS. Subsequent analysis of mitotic recombination events in PSL101 showed that the *URA3* allele was in coupling with *SUP4-o* on chromosome V.

MD457, a *spo11::NAT/spo11::NAT* derivative of PSL101, was constructed by mating the haploids MD454 and MD455. MD454 was constructed by transformation of PSL2 with a DNA fragment obtained by amplifying the *NAT*-containing plasmid pAG25 [6] with the primers SPO11NATF and SPO11NATR. The resulting nourseothricin-resistant transformant was checked by PCR amplification of genomic DNA with the primers VIII-63628 and nat-R. MD455, the other *spo11::NAT* haploid, was derived from the haploid MD416 by the same procedure. MD416 is a *URA3* derivative of PSL5 resulting from transformation of PSL5 with a *URA3*-containing DNA fragment generated by PCR amplification of genomic DNA of YJM789 with the primers URA3 US and URA3 DS.

PG311 is a *MATa/MATα::NAT* derivative of PSL101. This diploid was constructed by transforming PSL101 with a DNA fragment generated by PCR amplification of the plasmid pAG25 with the primers MATalpha NATF and MATalpha NATR. The resulting nourseothricin-resistant transformant was, as expected, sensitive to the alpha pheromone, capable of mating to a *MATα *haploid, and incapable of sporulation.

**Details of physical analysis of markers in sectored colonies.**

Of the approximately 100 sectored colonies analyzed, 12 were analyzed for all 34 polymorphic sites in both sectors; the PCR primers used in the analysis are in Table S2. For each marker, we usually observed one of four patterns: 1) the marker was heterozygous in both sectors (indicating that the RCO occurred centromere-distal to the marker), 2) the red sector was homozygous for the W303A form of the polymorphism and the white sector was homozygous for the YJM789 form of the polymorphism (indicating that the RCO occurred centromere-proximal to the marker), 3) the red sector was homozygous for the W303A form of the polymorphism and the white sector was heterozygous (indicating a gene conversion event in which the W303A-derived chromosome was a donor), and 4) the white sector was homozygous for the YJM789 form of the polymorphism and the red sector was heterozygous (indicating a gene conversion event in which the YJM789-derived chromosome was a donor).

For those sectored colonies that were not analyzed for all markers, we mapped the crossover and associated conversion tract (if any) by the following procedure. First, we examined the markers 57, 94, and 122 in both sectors; the numbers of the markers represent their approximate position (in thousands of base pairs) on chromosome V (SGD coordinates). This analysis allowed us to map the crossover within a single quadrant of the 120 kb interval. We subsequently refined the mapping within each quadrant using the additional heterozygous markers. Each crossover and gene conversion event was mapped to the highest degree of resolution possible with the 34 markers. Markers with unusual segregation patterns were re-checked. It should be noted that, in strains heterozygous for the *ura3* mutation, a sectored colony with red Ura^-^ and white Ura^+^ sectors were assumed to reflect a RCO between *CEN5* and *URA3*; the position of the RCO was confirmed by marker analysis.

**Supplementary Results and Discussion**

**Mitotic and meiotic conversion tract lengths.**

The maximum, minimum, and average mitotic conversion tract lengths for strains PSL100/PSL101, MD457, and PG311 are shown in Table S3-S5, respectively. Meiotic conversion tract lengths for PSL101 are shown in Table S6.

**Interpretation of recombination events in which the conversion tracts have an unexpected pattern of segregation in the sectored colonies.**

As discussed in the text, most (42 of 48) of the 3:1 or hybrid 3:1, 4:0 tracts had the pattern of marker segregation shown in Figure 4B. If the W303A-derived chromosome is the donor in the conversion event (the red chromatid in Figure 4B), we expected that the red sector would be homozygous for the converted marker and the white sector would be heterozygous; alternatively, if the YJM789-derived chromosome is the donor, we expected that the white sector would be homozygous for the converted marker and the red sector would be heterozygous. In 3 of 48 sectored colonies, we found a different pattern. One example is shown in the bottom part of Figure S1. In this example, although the W303A-derived chromosome is the donor in the conversion event, the red sector is heterozygous for the converted marker and the white sector is homozygous. This pattern can be explained as a consequence of the repair of two DSBs in which the repair of the first DSB is associated with a crossover with no conversion tract and the repair of the second has an associated conversion, but no associated crossover.

In the second class of exceptional sectored colonies (3 of 48 sectored colonies), the pattern of marker segregation indicated a crossover within the conversion tract (bottom part of Figure S2). This pattern can also be explained as repair of two DSBs resulting from a replicated broken chromosome. The repair of the first DSB is associated with a crossover and conversion of the marker centromere-distal to the DSB. The repair of the second DSB is not associated with a crossover, but results in conversion of a marker centromere-proximal to the DSB. It should be emphasized that there are other patterns of crossovers and conversions in G1 and G2 that could also explain the exceptional classes.

**Esposito model of mitotic recombination in G1**

As discussed in the main text, Esposito [7] concluded that a substantial fraction of spontaneous mitotic recombination was initiated in G1. In these experiments, a diploid was constructed that had non-complementing heteroalleles at the *TRP5* locus (*trp5-d* and *trp5-c*) and the heterozygous centromere-distal marker *ADE5*. The two main classes of sectored colonies diagnostic of a G1 event were: Class I (*ADE5/ADE5 TRP5/trp5-d* sector and *ade5/ade5 TRP5/trp5-d* sector) and Class II (*ADE5/ADE5 TRP5/trp5-c* sector and *ade5/ade5 TRP5/trp5-c* sector). For Class I events, since the *trp5-c* allele is not present on either homologue in either sector, this region of the *TRP5* gene has undergone a gene conversion equivalent to a 4:0 event; similarly, the *trp5-d* portion of the gene represents a 4:0 conversion in Class II.

Esposito [7] suggested that these results could be explained as a consequence of heteroduplexes formed in G1, followed by “patchy” repair of the resulting mismatches (repair of two adjacent mismatches using different strands as templates), and replication of the DNA molecules containing the resulting Holliday junction (Figure S3). Although we cannot exclude the Esposito model, we would expect the Holliday junction to be susceptible to resolvases rather than resolution by DNA replication. In addition, as described in the text, there is a single Holliday junction in the Esposito model, whereas the repair of meiotic DSBs in *S. cerevisiae* is associated with two adjacent Holliday junctions. Resolution of the double junction by replication would produce a double crossover rather than the single crossover required to make the distal marker homozygous. For these reasons, we prefer our model (Figure 8).

**Generation of long gene conversion tracts by BIR events**

As discussed in the text, one alternative model for generating very long conversion tracts involves two concerted cycles of BIR. This model is depicted in Fig. S4.

**References**

1. [Barbera MA](http://www.ncbi.nlm.nih.gov/sites/entrez?Db=pubmed&Cmd=Search&Term="Barbera MA"%5BAuthor%5D&itool=EntrezSystem2.PEntrez.Pubmed.Pubmed_ResultsPanel.Pubmed_DiscoveryPanel.Pubmed_RVAbstractPlus), [Petes TD](http://www.ncbi.nlm.nih.gov/sites/entrez?Db=pubmed&Cmd=Search&Term="Petes TD"%5BAuthor%5D&itool=EntrezSystem2.PEntrez.Pubmed.Pubmed_ResultsPanel.Pubmed_DiscoveryPanel.Pubmed_RVAbstractPlus) (2006) Selection and analysis of spontaneous reciprocal mitotic cross-overs in Saccharomyces cerevisiae. Proc Natl Acad Sci U S A 103:12819-24.

2. Thomas BJ, Rothstein R (1989) Elevated recombination rates in transcriptionally active DNA. Cell 56: 619-630.

3. Wei W, McCusker JH, Hyman RW, Jones T, Ning Y *et al.* (2007) Genome sequencing and comparative analysis of *Saccharomyces cerevisiae* strain YJM789. Proc Natl Aca. Sci U S A 104: 12825-12830.

4. Storici, F, Lewis LK, Resnick MA (2001) In vivo site-directed mutagenesis using oligonucleotides. Nat Biotech 19: 773-776.

5. Boeke JD, Lacroute F, Fink GR (1984) A positive selection for mutants lacking orotidine-5’-phosphate decarboxylase activity in yeast: 5-fluoro-oritic acid resistance. Mol Gen Genet 197: 345-346.

6. Goldstein AL, McCusker JH (1999) Three new dominant drug resistance cassettes for gene disruption in *Saccharomyces cerevisiae*. Yeast 15; 1541-1553.

7. [Esposito MS](http://www.ncbi.nlm.nih.gov/sites/entrez?Db=pubmed&Cmd=Search&Term="Esposito MS"%5BAuthor%5D&itool=EntrezSystem2.PEntrez.Pubmed.Pubmed_ResultsPanel.Pubmed_DiscoveryPanel.Pubmed_RVAbstractPlus) (1978) Evidence that spontaneous mitotic recombination occurs at the two-strand stage. Proc Acad Sci U S A 75:4436-40.
